# Macrophage-Mediated *In Vivo* Intracellular Crystallization of Aluminum Oxyhydroxide Adjuvant in Vaccine-Induced Granulomas

**DOI:** 10.1021/acsami.6c04089

**Published:** 2026-04-30

**Authors:** Estela Pérez, Marta Navarro, Alfonso Ibarra, Ignacio de Blas, Marta Pérez, Lluís Luján, Víctor Sebastián

**Affiliations:** a Department of Animal Pathology, University of Zaragoza, 177 Miguel Servet Street, Zaragoza 50013, Spain; b Institute of Nanoscience and Materials of Aragon (INMA), CSIC-University of Zaragoza, Mariano Esquillor Gómez Street I+D+i building, Zaragoza 50018, Spain; c Advanced Microscopy Laboratory, University of Zaragoza, Mariano Esquillor Gómez Street I+D+i building, Zaragoza 50018, Spain; d Agri-Food Institute of Aragon (IA2), University of Zaragoza, 177 Miguel Servet Street, Zaragoza 50013, Spain; e Department of Anatomy, Embryology and Genetics, University of Zaragoza, 177 Miguel Servet Street, Zaragoza 50013, Spain; f Department of Chemical and Environmental Engineering, University of Zaragoza, 3 María de Luna Street, Zaragoza 50018, Spain; g Networking Research Center on Bioengineering, Biomaterials and Nanomedicine (CIBER-BBN), 3-4 Monforte de Lemos Street, Madrid 28029, Spain

**Keywords:** gamma-alumina, intracellular crystallization, vaccine adjuvants, sheep, granulomas, crystalloid bodies, macrophages

## Abstract

Aluminum (Al) in the form of γ-aluminum oxyhydroxide (γ-AlOOH) nanoparticles has been used as a vaccine adjuvant for nearly a century. At injection sites, animals and humans develop granulomatous inflammation, in which macrophages may contain intracellular crystalline-like structures, so-called crystalloid bodies (CBs), morphologically distinct from the injected adjuvant. This raised the hypothesis that CBs may originate from the *in vivo* crystallization of γ-AlOOH within macrophages. To test this, six sheep received two subcutaneous doses of a γ-AlOOH-adjuvanted viral vaccine, while six controls received the same antigen without an adjuvant. All injection sites (AlOOH: *n* = 12; controls: *n* = 12) were examined by hematoxylin and eosin, modified aluminum hematoxylin, and lumogallion fluorescence. Al and CBs occurred exclusively in AlOOH-vaccinated tissues. The adjuvant stock and vaccine formulations were characterized *ex situ* using a combination of structural and imaging techniques. Four granulomas containing CBs, collected 133 days postinjection, were further examined using *in situ* imaging and crystallographic approaches. No crystalline structures resembling CBs were identified in the injected products; only γ-AlOOH nanoparticles were present. In post-mortem granulomas, macrophages contained Al-positive vacuoles filled with nanoparticle aggregates compatible with γ-AlOOH, often adjacent to CBs. Crystallographic analyses demonstrated that CBs were highly ordered γ-Al_2_O_3_ microcrystals, and advanced SEM-EDS with elemental mapping similarly distinguished γ-AlOOH nanoparticles and CBs as separate phases. These findings support an *in vivo* formation of γ-Al_2_O_3_ microcrystals from γ-AlOOH nanoparticles within macrophages under physiological conditions. To our knowledge, these findings provide evidence consistent with Al crystallization within mammalian cells and suggest that macrophage phagolysosomal conditions may represent a previously unrecognized biomimetic pathway for γ-Al_2_O_3_ formation. This finding may offer a biological model for the development of green and low-energy synthesis strategies for γ-Al_2_O_3_, with potential industrial applications as well as relevance to immunotoxicology, pathology, and cell biology in the context of vaccine adjuvants.

## Graphical abstract

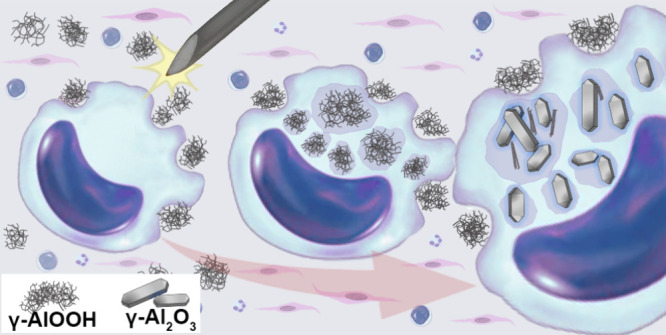

## 1 Introduction

Aluminum (Al) is the third most abundant element in the Earth crust, after silicon (Si) and oxygen (O), and is predominantly found in the form of oxide and silicate minerals, such as aluminosilicates.^1–3^ In its soluble ionic form (Al^3+^), Al is toxic to most organisms. Despite its abundance, no essential biological role has been identified for Al in any living organism.^1,3,4^ Its apparent biological inertness, combined with high toxicity, suggests that Al cycling in terrestrial ecosystems is largely governed by inorganic geochemical processes.^2,4^ The absence of genomic signatures or evidence of evolutionary exposure to Al supports the hypothesis that Al was locked in insoluble forms within the lithosphere, being inaccessible for the biosphere at critical points of biochemical evolution.^1,2^ In the modern era, anthropogenic indirect and direct influences (particularly acid rain and unsustainable agricultural practices) have acidified soils and water systems, enhancing the mobilization of Al from mineral reserves and increasing its environmental bioavailability.^1–3^ Since the late 1980s, Al mobilization has been further driven by large-scale industrial extraction from bauxite ores, fueled by global technological demand due to Al distinctive chemical, mechanical, and electrical properties.^1,5^ This demand is expected to potentially increase further.^6^

Pseudoboehmite, a form of aluminum oxyhydroxide (AlOOH) composed of smaller crystallites than boehmite (γ-AlOOH), exhibits high surface area and porosity.^7,8^ Aluminum oxyhydroxides exist in several related forms, including boehmite (γ-AlOOH) and pseudoboehmite, which differ primarily in crystallinity and hydration state. In contrast, aluminum trihydroxides (Al­(OH)_3_), including gibbsite (γ-Al­(OH)_3_) and bayerite (α-Al­(OH)_3_), as well as gamma-alumina (γ-Al_2_O_3_), represent distinct structural phases. These properties support the widespread industrial use of pseudoboehmite as a catalyst, adsorbent, surfactant, and component in Al-based ceramics.^7,8^ It also serves as a primary precursor to γ-Al_2_O_3_, the metastable alumina polymorph with the greatest industrial and technological relevance, although its synthesis typically requires high temperatures, expensive reagents, and complex equipment.^6,7,9–11^ Gamma-alumina is widely used as a catalyst, catalyst support, and adsorbent in the automotive, petrochemical, electronics, and ceramics industries.^7,9^ Compared to other precursors such as boehmite or aluminum trihydroxides (Al­(OH)_3_), pseudoboehmite enables the production of high-surface-area γ-Al_2_O_3_ with lower energy input.^7,12^ Importantly, one of the most longstanding, impactful, and often overlooked uses of pseudoboehmite is its role as a potent immunostimulant in Al-based adjuvants used in both human and veterinary vaccines.^13,14^

Pseudoboehmite nanoparticles, from now on referred to as an AlOOH adjuvant, have been the most widely used immunostimulant in commercial vaccines for nearly a century.^13,15^ However, the persistent mislabeling of this material as Al hydroxide has led to longstanding confusion with true Al­(OH)_3_ such as bayerite and gibbsite.^13,14,16^ The so-called Al hydroxide adjuvant is composed of pseudoboehmite nanoparticles in the form of fine nanorods or needles that are densely entangled into dynamic and polydisperse microaggregates measuring between 1 and 100 μm, which constitute the functional immunostimulatory unit of the adjuvant.^13–15,17^ Although these nanoparticles are described as measuring approximately 4 × 2 × 10 nm, their strong tendency to aggregate often severely hinders accurate characterization, even after sonication.^13,18^ These primary nanoparticles are defined by their small crystallite size and high surface area, features that confer substantial porosity to their microaggregates.^13–15,17^ This results in a high protein adsorption capacity that exceeds that of crystalline Al­(OH)_3_ and considered to play an important role in their immunostimulatory activity as an adjuvant.^13,16^

Although the precise mechanisms of AlOOH adjuvanticity remain unclear, it induces local cytokine and chemokine release that promotes recruitment of inflammatory cells, particularly macrophages, and the formation of granulomas.^19–21^ Antigen adsorption onto AlOOH nanoparticles is thought to enhance antigen retention and cellular uptake.^22–24^ However, histiocytic cells avidly phagocytose AlOOH nanoparticles *in vivo* and *in vitro*, recognizing them as foreign bodies regardless of antigen presence.^19,21,24^ Macrophages can internalize large amounts of AlOOH while maintaining apparent viability, and within phagolysosomes, the particles reorganize into larger, needle-like aggregates approximately 54 × 8 nm.^19,25^ In parallel, we have consistently observed homogeneous, hyaline, cigar-shaped microstructures with sharply defined borders within macrophages in ovine granulomas, which we designated crystalloid bodies (CBs) due to their apparent, though initially unconfirmed, crystallinity.^19^ These structures are routinely observed in both diagnostic and experimental evaluations of sheep granulomas^19,20,26^ and were recently reported in a feline injection-site sarcoma.^26^ Comparable formations have also been described in porcine granulomas.^27^ In humans, similar structures were sporadically reported between the 1960s and 1980s under various terms, including hyaline bodies, aluminum hydroxide inclusions, and angular deposits.^28–31^ Their precise nature and impact on the granulomatous microenvironment remain unclear, but their consistent association with AlOOH-induced inflammation, whether from vaccines or adjuvant-only injections, suggests the hypothesis that they form *in vivo* during the inflammatory response.^19,20,26^ This was reinforced by our recent findings: although the AlOOH adjuvant was extensively characterized before formulation with an inactivated virus, CBs appeared consistently in all postvaccination granulomas but were not present in the original adjuvant preparation.^32^

Using a multidisciplinary approach, this study characterizes Al-based CBs in sheep granulomas from the aforementioned trial in terms of morphology, composition, and mineralogy, aiming to confirm their crystalline nature and identify the *in situ* Al phase. Both the AlOOH adjuvant nanoparticles and the vaccines used to induce granulomas were extensively characterized *ex situ* to rule out prior CB formation. Adjuvant nanoparticles within macrophages were also analyzed due to their potential role as nucleation centers. To the best of our knowledge, this is the first study to provide evidence consistent with an *in vivo* and intracellular crystallization of an Al compound in mammalian cells. Remarkably, the observations are consistent with the formation of γ-Al_2_O_3_ crystals from the AlOOH adjuvant within macrophages under physiological conditions and at temperatures far below those required for conventional synthesis. Given the toxicity of elemental Al^1^ and the natural rarity of Al_2_O_3_ phases (which typically require high-temperatures and pressures),^10^ these findings suggest potentially novel pathways for γ-Al_2_O_3_ formation. Although far from immediate practical application, this discovery opens long-term prospects for biomimetic, low-energy approaches to γ-Al_2_O_3_ synthesis. If the underlying mechanisms are elucidated, it could inspire alternative routes operating under milder conditions, with the potential to reduce the energy demand and environmental impact of conventional high-temperature processes, while raising new questions about host–Al interactions in pathology, immunotoxicology, and cell biology. A schematic illustration of the conceptual framework of this study, highlighting its materials science perspective, is presented in Figure 1.

**Figure 1:**
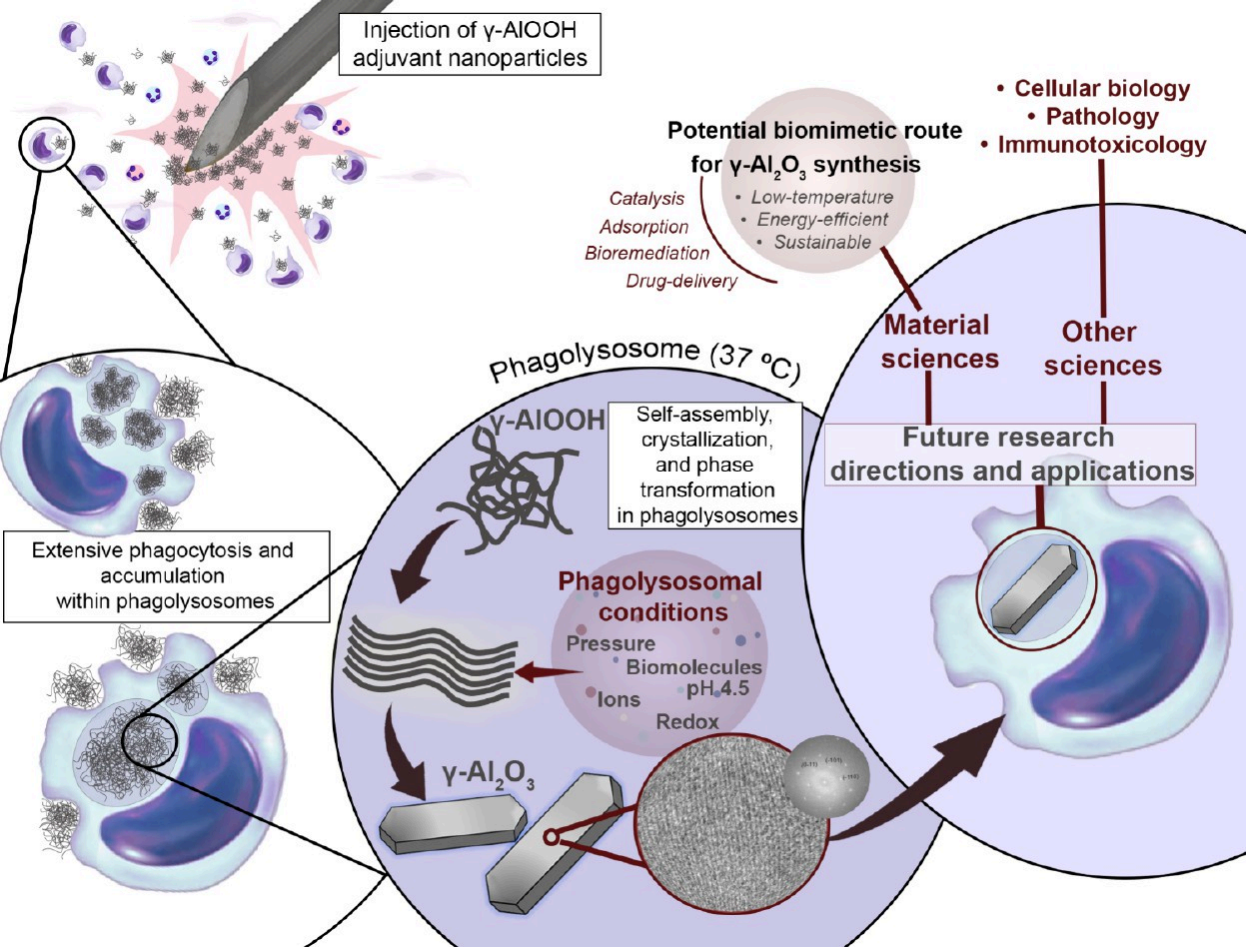
Schematic illustration of the conceptual framework of this study, showing the *in vivo* transformation of γ-AlOOH adjuvant nanoparticles into γ-Al_2_O_3_ microcrystals within macrophages. The diagram highlights key processes, including macrophage uptake, phagolysosomal conditions, and intracellular self-assembly and phase transformation. From a materials science perspective, this phenomenon suggests a potential low-temperature, aqueous route for γ-Al_2_O_3_ formation, with broader implications across related scientific fields.

## 2 Results

### 2.1 Physicochemical Characterization of the Injected AlOOH Adjuvant and Vaccine

An aliquot of the AlOOH adjuvant (Adjuval), collected directly from the commercial stock solution prior to homogenization and diluted in PBS (0.1 mg mL^–1^), was subjected to physicochemical characterization. The preparation consisted of small particles with a median (±interquartile range) of 2.3 ± 0.6 nm (*n* = 110), which were mildly laminated and often curved, forming intertwined aggregates, as observed by TEM and HAADF-STEM (Figure 2A,B). EDS confirmed that the particles were primarily composed of Al and O (Figure 2C). A diluted adjuvant sample in PBS at the concentration used in the vaccine (6 mg mL^–1^) showed large aggregates of nanoparticles composed of Al and O under SEM, covered by crystalline structures consisting solely of Cl and Na, consistent with halite-like minerals (Figure 2D). In contrast, SEM analysis of the commercial AlOOH adjuvant undiluted in PBS (30 mg mL^–1^) revealed only nanoparticle aggregates of Al and O, with no crystals detected (Figure 2E). Sulfur was detected in the adjuvant regardless of PBS dilution, whereas phosphorus, chlorine, and sodium were detected predominantly in the PBS-diluted sample. Laser diffraction analysis (LDA) of the adjuvant aggregates at 6 mg mL^–1^ in suspension revealed a trimodal size distribution, with median sizes of 8.9 μm for the first population, 34.4 μm for the second, and up to 194.8 μm for the third population (Figure 2F). Additional LDA data are listed in Table S1. XRD analysis of the undiluted adjuvant identified a single phase of boehmite (γ-AlOOH, ICDD #PDF-01–072–03559) but with extensive broad peaks, especially the 020-diffraction peak (Figure 2G).

**Figure 2:**
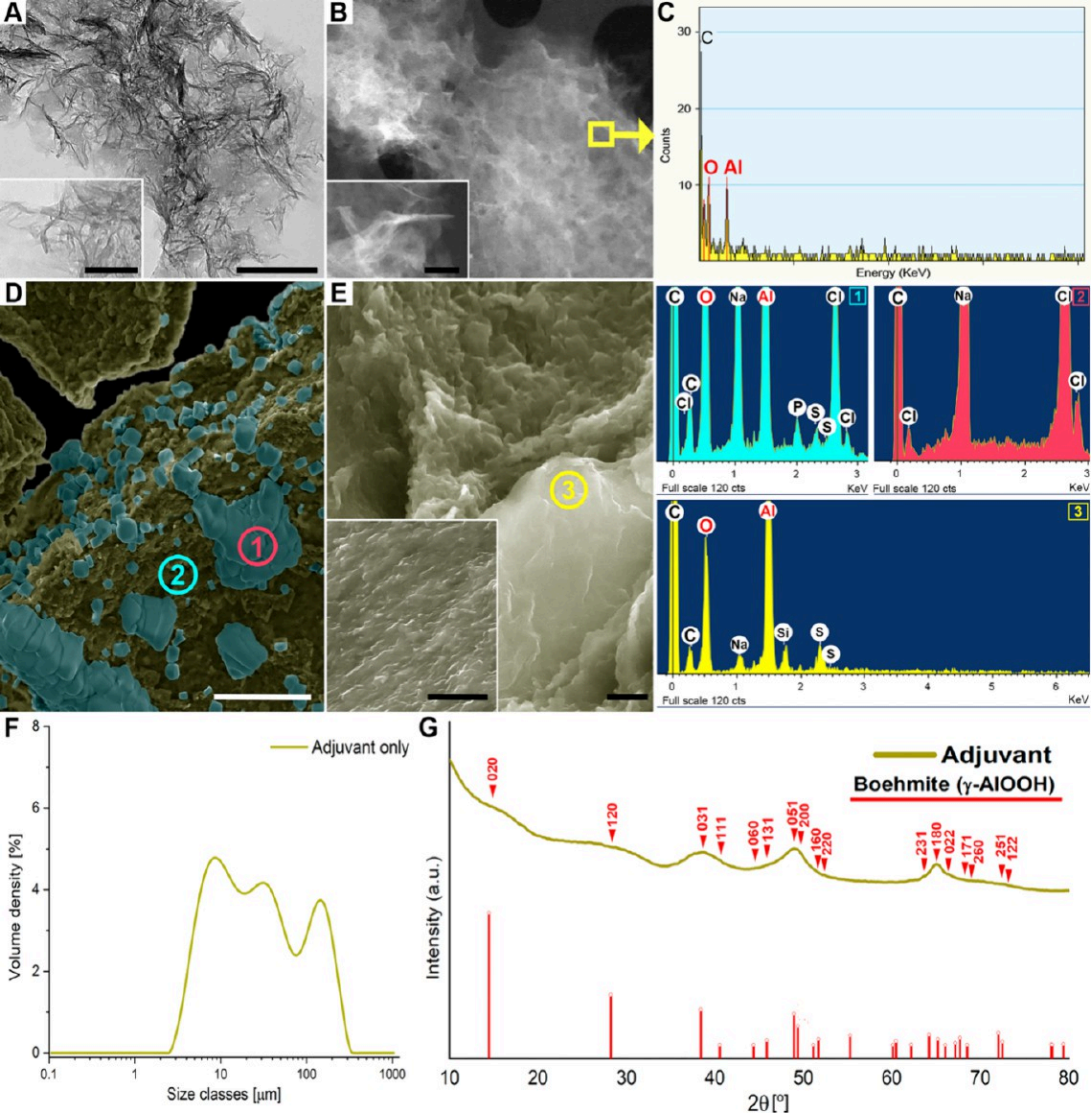
Physicochemical characterization of aluminum (Al) oxyhydroxide adjuvant (AlOOH) used for sheep vaccination. (A) TEM image of the adjuvant nanoparticles. Bar: 200 nm. Insert bar: 50 nm. (B) HAADF-STEM image of adjuvant nanoparticles, where Z-contrast is observed. Bar: 100 nm. Inset bar: 50 nm. (C) EDS of nanoparticles in STEM. The carbon peak originates from the grid support film. (D) SEM image of a diluted sample at 6 mg mL^–1^ in PBS. SEM images were digitally colorized to highlight adjuvant nanoparticle aggregates (green) covered by crystals (blue); colors are for visualization only and do not represent elemental composition. EDS of aggregates (1) revealed Al and oxygen (O), whereas analysis of crystals (2) detected only chlorine (Cl) and sodium (Na). (E) SEM of the undiluted adjuvant (without PBS). No crystals observed; EDS (3) shows Al and O, like spectrum 1. (F) Laser diffraction analysis of 6 mg mL^–1^ samples (*n* = 3) showing three distinct aggregate populations or modes (M: M1, M2, and M3). Data indicate the mean ± standard error of the mean. D­[4,3]: volume-weighted mean diameter; Dv50: 50% (median) of the cumulative volume distribution (%). (G) XRD of the undiluted adjuvant. Diffraction peaks match a boehmite phase (γ-AlOOH, ICDD #PDF-01–072–03559).

The composition, Al content, and formulation procedures of the AlOOH vaccine used to induce granulomas in sheep are detailed in the Experimental section. The same vaccine vial used for sheep immunization was analyzed by SEM at both undiluted (Figure 3A) and 1:5 (Figure 3B) and 1:10 dilutions (Figure 3C) in PBS. Similar to the adjuvant diluted in PBS alone, halite-like crystals were observed in both undiluted and diluted samples. In the undiluted vaccine, these crystals measured 4.4 ± 2.4 μm (mean ± SD; *n* = 50). Although they contained Al and O, their proportions were minimal compared to those of chlorine and sodium (Figure 3D). In contrast, surrounding aggregates, resembling those in the adjuvant alone, exhibited a higher proportion of Al and O relative to those of chlorine and sodium (Figure 3E). Consistently, XRD analysis revealed a mixture of phases, including the broad diffraction peaks previously described for the boehmite adjuvant alongside peaks from crystalline phases characteristic of halite (COD ID-1000041) (Figure 3F). Vaccine aggregates in suspension showed a monomodal distribution of polydisperse aggregates with a mean ± standard error of 41.3 ± 3.0 μm (Figure 3G).

**Figure 3:**
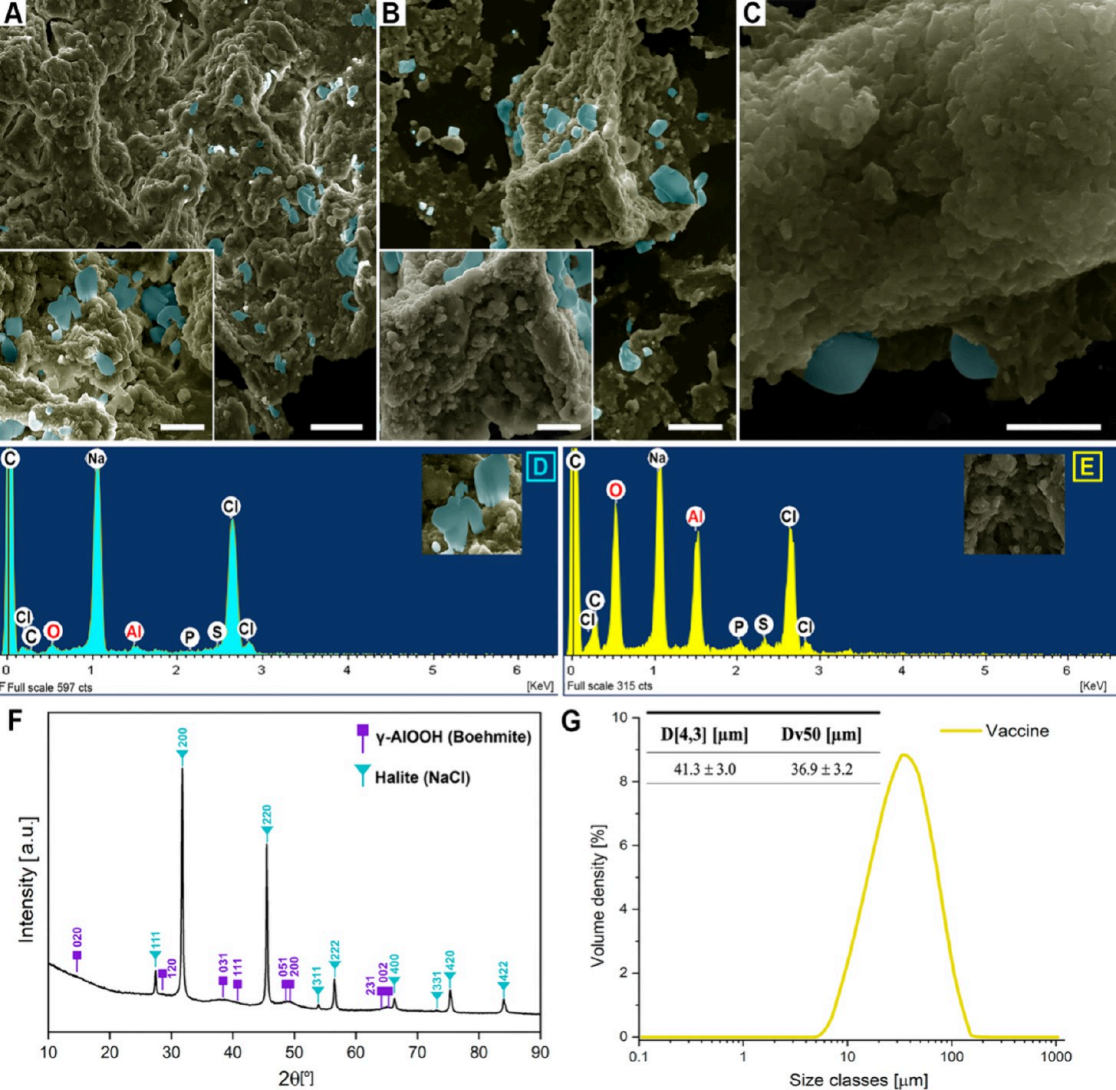
Physicochemical characterization of the vaccine containing aluminum (Al) oxyhydroxide adjuvant (AlOOH) used for sheep vaccination. (A) SEM image of an undiluted vaccine. Scale bar: 30 μm; inset scale bar: 5 μm. (B) SEM image of vaccine diluted 1:5 in PBS. Scale bar: 30 μm; inset scale bar: 5 μm. (C) SEM image of vaccine diluted 1:10 in PBS. Scale bar: 1 μm. SEM images were digitally colorized to highlight adjuvant nanoparticle aggregates (green) covered by crystals (blue); colors are for visualization only and do not represent elemental composition. (D) EDS of crystals (blue) detected in all vaccine samples. (E) EDS of adjuvant aggregates (yellow) was detected in all vaccine samples. (F) XRD analysis of vaccine showed a mixture of nanostructured boehmite (γ-AlOOH, ICDD #PDF-01–072–03559) and halite crystals (COD ID-1000041). (G) Laser diffraction analysis of vaccine samples (*n* = 3) showing a monomodal distribution of aggregate populations. Data indicate mean ± standard error of the mean. D­[4,3]: volume-weighted mean diameter; Dv50: 50% (median) of the cumulative volume distribution (%).

Al_2_O_3_ crystals, or crystals with similar characteristics, were not detected in either the adjuvant (Figure 2) or the vaccine (Figure 3), at any concentration tested, by any of the analytical techniques used.

Overall, the injected adjuvant and vaccine consisted of aggregated γ-AlOOH nanoparticles with no evidence of γ-Al_2_O_3_ or similar crystalline phases detected under any of the experimental conditions.

### 2.2 Macroscopic and Histopathological Analyses

Macroscopic nodular inflammatory reactions developed exclusively at AlOOH injection sites (10/12 injections, six sheep). The nodules were well-demarcated, yellow to tan in color, and located within the adipose panniculus, often adherent to the underlying muscle fascia. Half of the found nodules displayed central caseous necrosis upon sectioning (Figure 4A,B). Histologically, necrosis was evident and accompanied mineral calcium deposits, confirmed by von Kossa and Alizarin Red S staining (Figure 4C,D). Microscopically, lesions were dominated by multinucleated macrophages with voluminous granular cytoplasm, occasional epithelioid macrophages, and scarce neutrophils and lymphocytes, as shown in (Figure 5A–C). Macrophage nuclei remained largely viable but were often displaced peripherally by cytoplasmic vacuoles and granules (Figure 5B,C). This eccentric distribution of vacuoles typically produced a clear central area within the cytoplasm.

**Figure 4:**
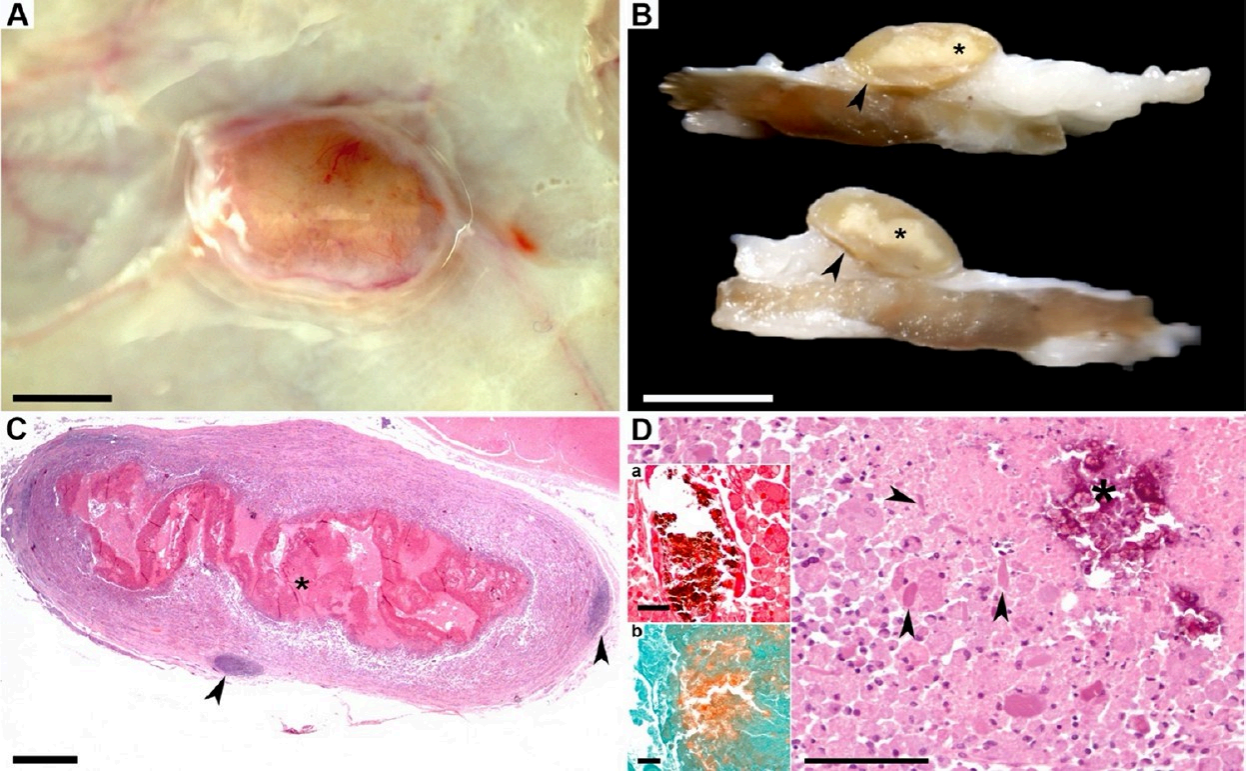
Granuloma induced by an AlOOH-adjuvanted vaccine in the subcutaneous tissue of a sheep. (A) Macroscopic view of the lesion. Bar = 0.5 mm. (B) Cross sections of granulomas (arrowheads) showing central caseous necrosis (asterisks) in fixed specimens. Bar = 1 cm. (C) Subgross image. A well-demarcated nodular inflammatory lesion is evident, featuring a large central area of necrosis (asterisk) and peripheral tertiary lymphoid aggregates (arrowheads). HE, bar: 1 mm. (D) The granuloma is composed almost exclusively of numerous granular macrophages undergoing necrosis toward the center of the lesion. During this process, eosinophilic intracellular crystalloid bodies are released into the extracellular space within the necrotic region (arrowheads). HE, bar: 100 μm. Basophilic deposits within the necrotic core (asterisks) are consistent with calcium salts, as indicated by their brown to black staining with von Kossa (insert a) and orange staining with Alizarin Red S (insert b). Both insert bars: 50 μm.

**Figure 5:**
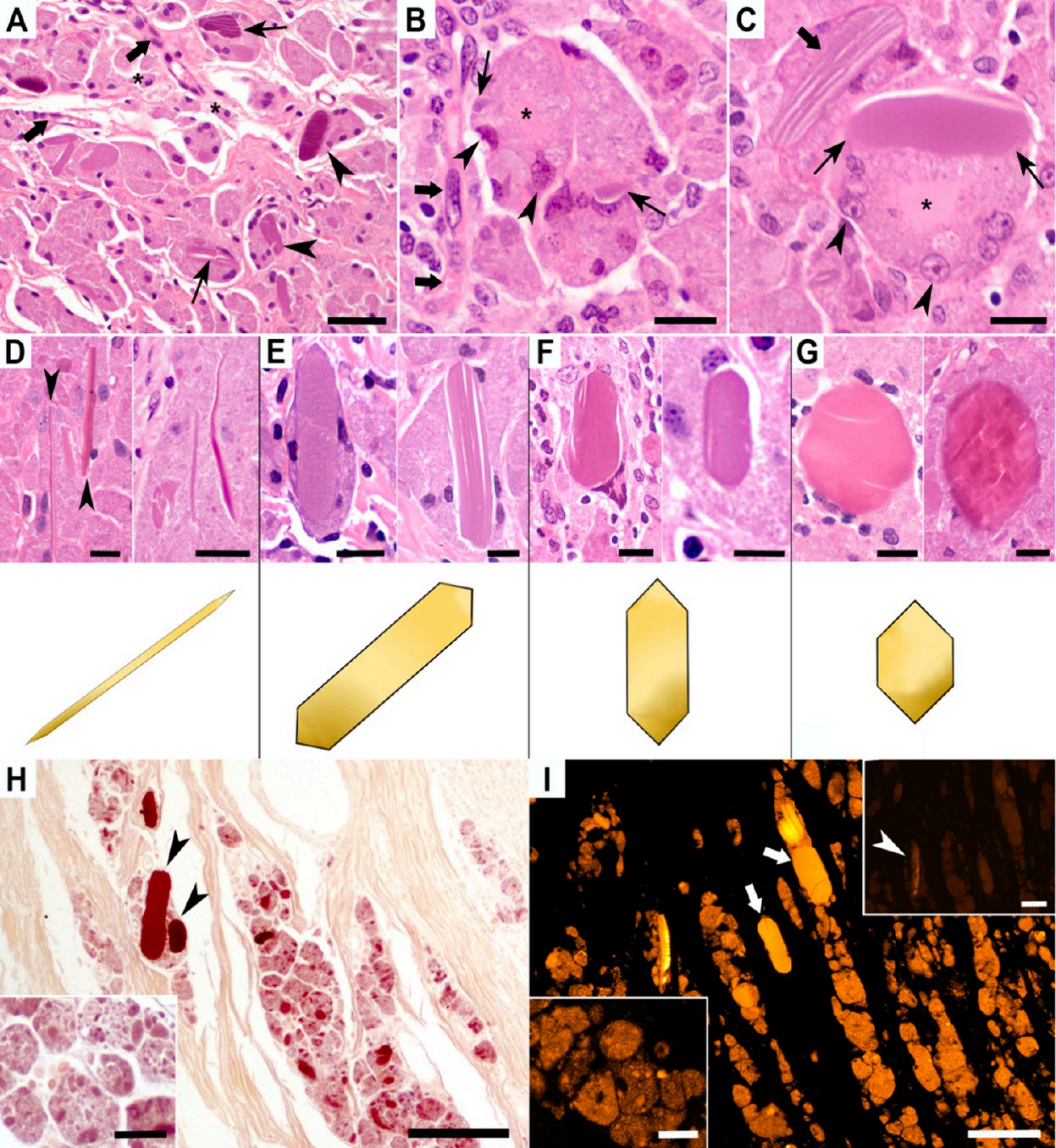
Histopathology of macrophages and crystalloid bodies (CBs) in subcutaneous granulomas induced by aluminum oxyhydroxide (AlOOH)-adjuvanted vaccines in sheep. (A) Granulomas mainly consisted of multinucleated macrophages with a granular cytoplasm and intracellular CBs eccentrically displacing nuclei (arrowheads). CBs exhibited variable morphologies, with straight edges and a homogeneous hyaline to amphophilic appearance; fine longitudinal fissures were frequent (thin arrows). Scattered epithelioid macrophages (asterisks) and vessels with hypertrophic endothelium (thick arrows) were occasionally observed. HE, bar: 50 μm. (B, C) At higher magnification, macrophages contained eccentric nuclei displaced by vacuoles with particulate material (arrowheads), leaving central clear zones (asterisks). Early hyaline CBs with peripheral granules (thin arrows), mature CBs with longitudinal fissures (thick arrows), and predominantly viable nuclei with occasional pyknosis were observed. HE, bars: 20 μm. (D–G) CB morphological variants: thin needles (D), long hexagonal rods of high aspect ratio (AR) (E), short hexagonal rods of low AR (F), and rounded hexagons (G). HE, bar: 15 μm. (H) Modified Al-hematoxylin (MAH) staining shows strong cytoplasmic (granular) and CB positivity (black arrowheads) versus negative connective tissue (yellow background). Inset: granular cytoplasmic pattern. MAH, bars: 50/25 μm. (I) Lumogallion fluorescence shows intense CB signals (thick white arrows) and granular cytoplasmic fluorescence. Inset (lower left): granular cytoplasmic pattern. Inset (upper right): consecutive negative control section with only mild CB autofluorescence (white arrowhead). Lumogallion, bars: 100/25/50 μm.

CBs were abundant, mostly intracellular within macrophages and sometimes extracellular in necrotic foci (Figure 4D). They showed sharp-edged morphologies, hyaline to amphophilic appearance, frequent longitudinal fissures, and an average size of 31.8 × 10.8 μm, with an aspect ratio of ∼2.9. Predominant forms were hexagonal rods and thin needles, while rounded hexagons were uncommon (Figure 5D–G).

Al localization was confirmed by modified hematoxylin-aluminum (MAH) staining and lumogallion fluorescence, both showing granular cytoplasmic deposits with increased positivity in CBs for both (Figure 5H–I). Control animals vaccinated with nonadjuvanted virus (no AlOOH) showed no positive staining at injection sites.

Modified hematoxylin-aluminum (MAH) staining demonstrated granular cytoplasmic Al deposits within macrophages with particularly strong reactivity in CBs (Figure 5H). Likewise, lumogallion fluorescence showed a comparable granular distribution with markedly increased signal intensity in CBs (Figure 5I). Both MAH and lumogallion staining were negative at injection sites from animals that received the nonadjuvanted virus, used as Al-free negative controls.

Granulomatous lesions were consistently observed at AlOOH injection sites and were characterized by macrophage-dominated inflammation, with abundant intracellular aggregates and CBs showing Al positivity.

### 2.3 Electron Microscopies, Energy-Dispersive X-ray Spectroscopy (EDS), and Crystallographic Analyses

SEM with backscattered electron detecting (BSED) analysis revealed granuloma architecture more distinctly than the Everhart–Thornley detector (ETD), sharply delineating macrophages with granular cytoplasm and CBs (Figure 6A–C). Cytoplasmic granules corresponded to heterogeneous aggregates of low to moderate signal intensity containing Al (Figure 6B–D). Nuclei (*n* = 4) consistently lacked detectable Al, phosphorus, or nitrogen, serving as internal negative controls (Figure 6D,E). CBs with distinct morphologies appeared as prominent structures within macrophages, displaying strong electron signals and clear Al peaks by EDS (Figure 6F–K).

**Figure 6:**
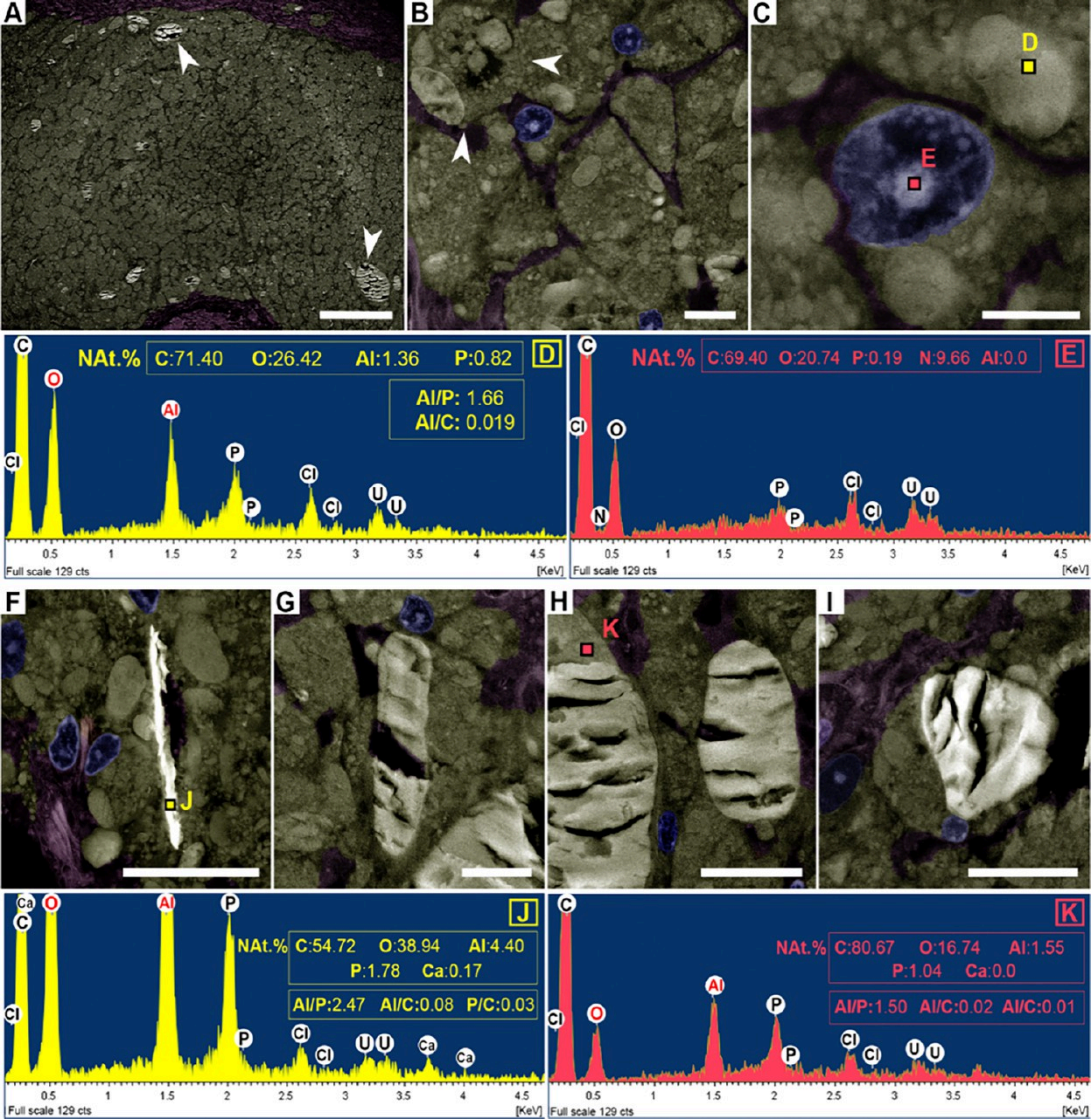
SEM with a backscattered electron detector (BSED) and EDS analyses of subcutaneous granulomas induced by aluminum oxyhydroxide (AlOOH)-adjuvanted vaccines in sheep (resin-embedded semithin section). SEM images were digitally colorized to highlight macrophages (golden-yellow hue), connective tissue (purple), and nuclei (blue); colors are for visualization only and do not represent elemental composition. NAt. % indicates normalized atomic percentage, excluding chlorine (Cl), uranium (U), and other trace elements derived from processing. (A) Granular macrophages with CBs of high signal intensity (arrowheads). Bar = 200 μm. (B) Macrophages with both cytoplasmic aggregates (asterisks) and CBs (arrowheads) of variable sizes. Bar = 10 μm. (C, D) Aggregates containing Al and O, together with C and P. Cl and U originate from the resin and staining. (E) Nucleus lacking detectable Al; C expected in organic tissue; Cl and U from resin and staining, respectively. (F–I) CBs of distinct shapes and intensities with fissures. Bars = 20 μm. (J) EDS spectrum of high-signal-intensity CBs (F), showing Ca. (K) EDS spectrum of lower-signal-intensity CBs (H), without calcium.

SEM-EDS point analyses detected Al and P in all CBs and adjuvant aggregates but not in the surrounding interstitial or stromal tissue, while Ca was present at low levels in a subset of CBs (4/19; 21%) (Figure 7A–C). O and C were consistently detected in CBs, aggregates, and adjacent tissue. Normalized atomic (NAt.%) and weight (NWt.%) percentages were obtained after excluding processing-derived contaminants (e.g., Cl, U, and Os) and proportionally recalculating the remaining elemental fractions to 100%. The complete unnormalized elemental compositions are provided in Table S2. CBs displayed significantly higher Al:C ratios than either adjuvant aggregates (*p* = 0.04) or surrounding tissue (*p* < 0.001) (Figure 7D), whereas Al:P ratios were similar between CBs and aggregates (Figure 7E, Table S3). In contrast, P/C ratios were significantly higher in CBs than in aggregates (*p* = 0.035) (Figure 7F). Correlation analysis showed that, within CBs, Al:C, Al:P, and P:C ratios were all positively and significantly correlated (*p* < 0.05), while in adjuvant nanoparticles only Al:C and P:C displayed a significant bidirectional correlation (*p* = 0.023), with Al:P showing no association with the other ratios (Table S4). CBs with higher electron signal intensity (*n* = 4) appeared to exhibit greater atomic levels of Al and P relative to C (Figure 6J,K), and comparison with low-intensity CBs (*n* = 15) confirmed significantly higher Al:C and P:C ratios in the high-intensity group (*p* = 0.001 and *p* = 0.006, respectively), with no differences in Al:P (Table S5). Among low-intensity CBs, Al:C and P:C ratios were strongly correlated (*p* < 0.001), while Al:P showed no correlation with either ratio (Table S6). Al:O atomic ratios tended to be slightly higher in CBs than in aggregates, though differences were not statistically significant (Table S3). Overall, measured Al:O ratios were approximately 1 order of magnitude lower than the theoretical stoichiometries expected for γ-Al_2_O_3_ (≈0.67)^33^ and γ-AlOOH (≈0.50),^34^ with CBs showing a mean ± standard deviation of 0.06 ± 0.03 and adjuvant aggregates showing 0.05 ± 0.01.

**Figure 7:**
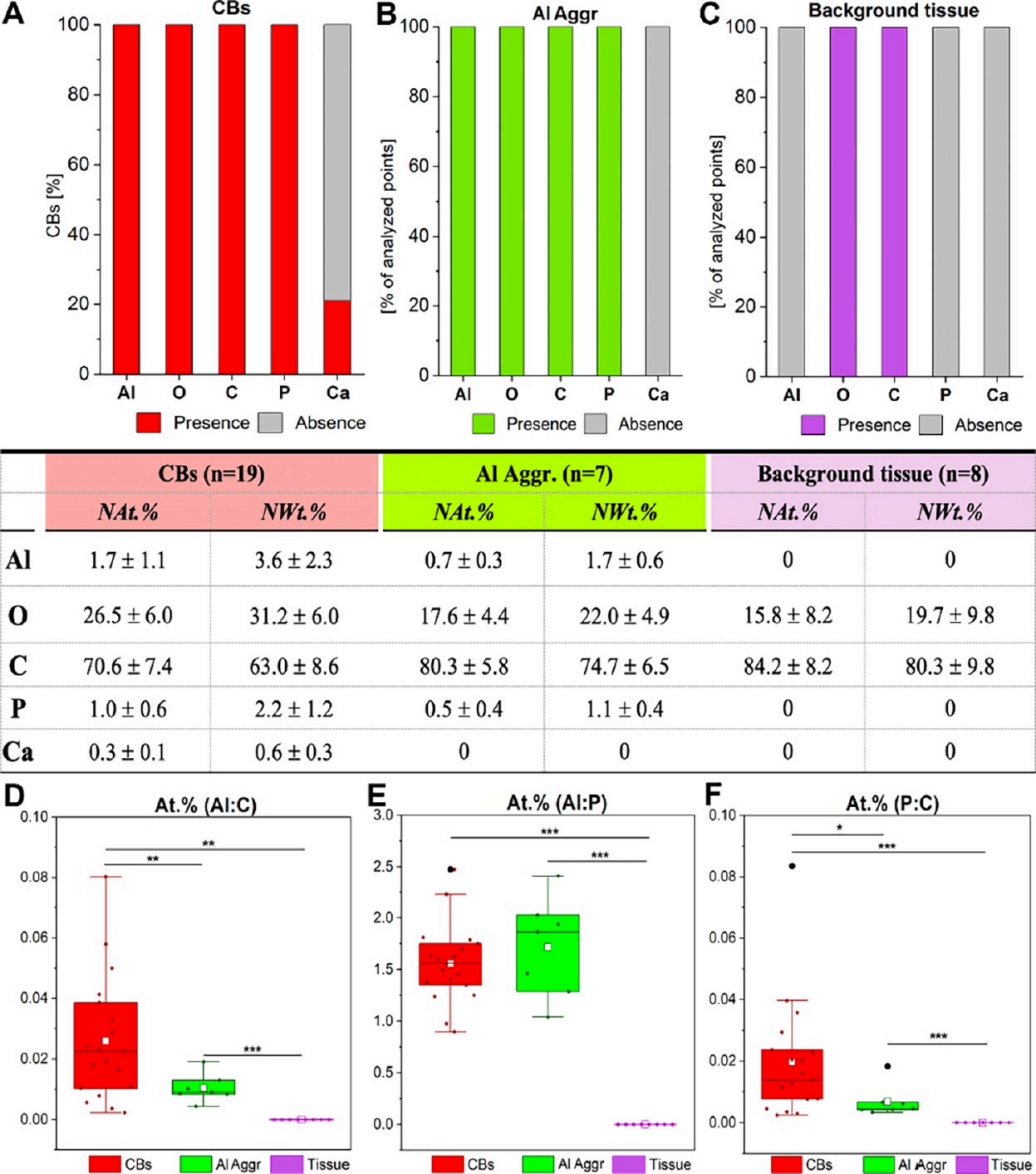
Elemental composition and ratios from SEM-EDS analyses of subcutaneous granulomas induced by aluminum oxyhydroxide (AlOOH)-adjuvanted vaccines in sheep. Elemental ratios (Al:C, Al:P, and P:C) are used as the primary comparative metrics across distinct microanatomical areas within granulomas. (A–C) Detection frequencies (bar graphs) and normalized quantities (table) of major elements identified in crystalloid bodies (CBs), aluminum aggregates (Al aggr.), and surrounding tissue. NAt.% and NWt.% represent normalized atomic and weight percentages, excluding chlorine (Cl), uranium (U), and other trace elements introduced during processing. (D–F) Comparative ratios of aluminum to carbon (Al:C), aluminum to phosphorus (Al:P), and phosphorus to carbon (P:C) across CBs, Al aggr., and surrounding tissue. Boxplots show interquartile ranges with medians (horizontal lines), mean (white squares), and whiskers indicating minimum and maximum values; outliers are shown as black dots. *p < 0.05, **p < 0.01, ***p < 0.001.

Most CBs appeared homogeneous under BSED (Figure 8A,B), although one showed surface heterogeneity detectable only with BSED (Figure 8A,C). Areas of higher signal corresponded to greater Al and P relative to C, while adjacent regions of lower intensity contained proportionally more C (Figure 8D).

**Figure 8:**
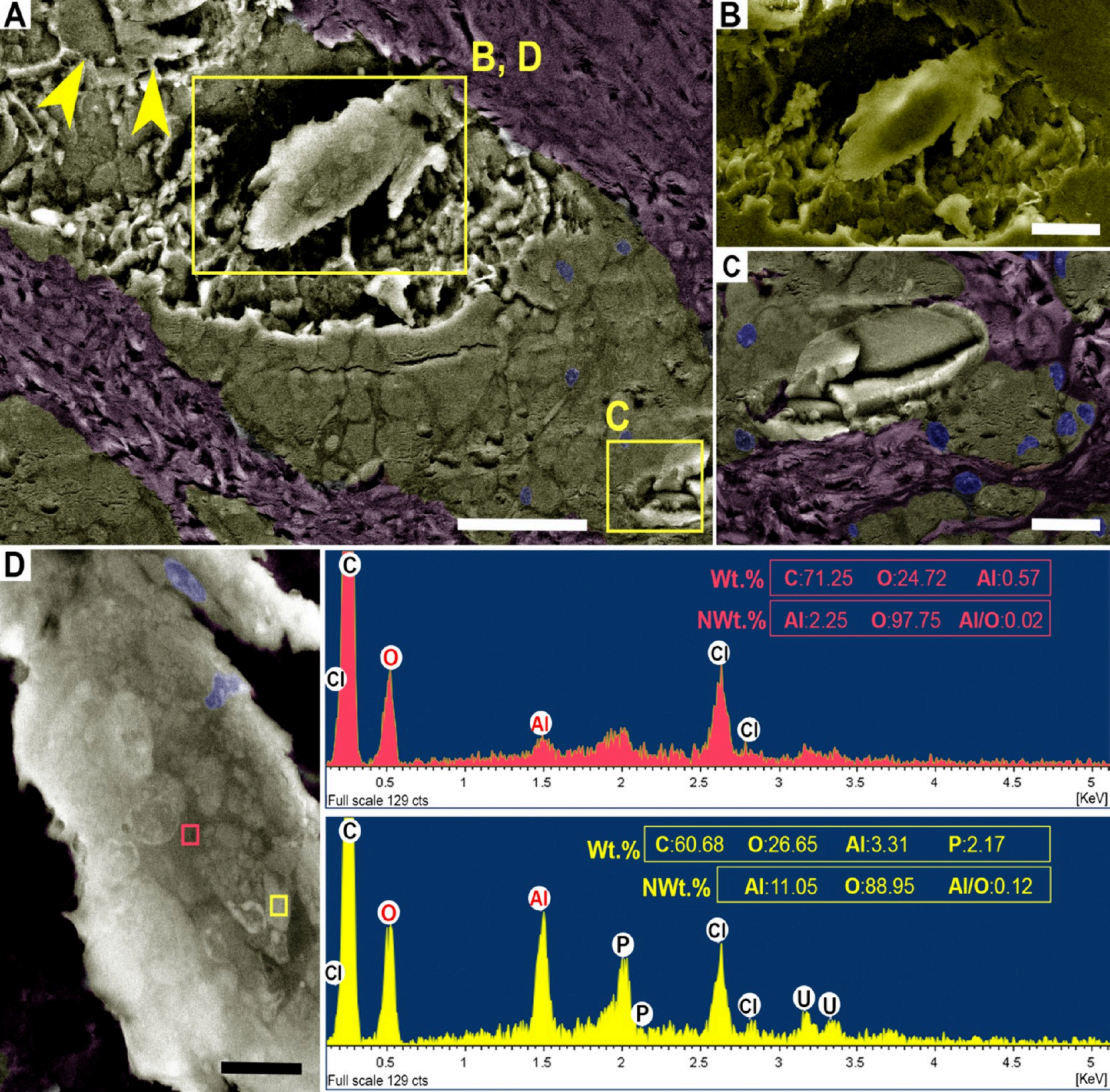
Additional SEM and EDS analyses of crystalloid bodies (CBs) in subcutaneous granulomas induced by aluminum oxyhydroxide (AlOOH)-adjuvanted vaccines in sheep (resin-embedded semithin section). SEM images were digitally colorized to highlight macrophages (golden-yellow), connective tissue (purple), and nuclei (blue); the colors are for visualization only and do not represent elemental composition. NAt.% refers to normalized atomic percentages, excluding chlorine (Cl), uranium (U), and other processing-related trace elements. (A) CBs (yellow boxes) were located within macrophage-rich areas. Some CBs show heterogeneous surface composition (large box), whereas others appear homogeneous (small box and arrowheads). Backscattered electron detector (BSED) image. Bar = 50 μm. (B–D) Higher-magnification views of homogeneous (B) and heterogeneous CBs (C–D). The CB marked in panel A (large box) imaged with an Everhart–Thornley Detector (ETD) does not reveal heterogeneity (C). In heterogeneous CBs (D), regions of higher signal intensity (yellow box) contained greater Al and phosphorus (P) relative to carbon (C) compared with adjacent lower-intensity areas (pink box). Bars: B–C = 20 μm; D = 10 μm.

Macrophages were the predominant cell type observed under TEM and HAADF- STEM imaging in all granulomas analyzed (n = 4) (Figure 9A,B), with occasional capillaries, neutrophils, and fibroblasts (Figure S1). Intracellular aluminum aggregates appeared as multiple membrane-bound vacuoles that displaced the nuclear envelope and caused nuclear deformation. These vacuoles contained laminated, needle-like nanoparticles with a median (±interquartile range) thickness of 2.3 ± 0.8 nm (*n* = 110) (Figure 9A–E), which were significantly thicker (*p* < 0.001) than the aluminum oxyhydroxide (AlOOH) nanoparticles characterized *ex situ* in the absence of the inactivated viral antigen from the vaccine. These appeared to be electron-dense under TEM and showed higher intensity under HAADF-STEM due to the inherent Z-contrast of high-angle annular dark-field imaging. Within vacuoles, nanoparticles showed variable aggregation, from dense clusters (Figure 9C–E). Small free aggregates of similar needles were also detected in the cytosol (Figure 9F,G). EDS analyses confirmed that both intravacuolar and cytosolic aggregates consisted predominantly of Al and O at all examined locations (6/6) (Figure 9H,I and Figure S2, respectively), whereas no detectable Al was observed in the nuclear regions (2/2). Crystallographic characterization of AlOOH nanoparticles was unfeasible, as they were embedded within the surrounding organic matrix and only visible in stained sections, resulting in a signal-to-noise ratio too low for reliable SAED or FFT pattern acquisition. In stained ultrathin sections obtained by room-temperature ultramicrotomy, most CBs were not preserved and appeared as void-like spaces corresponding to their expected morphology, likely due to their displacement during sectioning (Figure S3). Occasionally, CBs were retained within folds of unstained ultrathin sections prepared by cryo-ultramicrotomy, where they appeared as electron-dense structures composed predominantly of Al and O (7/7) (Figure 9J). TEM-EDS analyses of CBs and Al nanoparticle aggregates revealed elemental compositions consistent with SEM-EDS results, primarily detecting Al, C, O, phosphorus, and calcium in both structures (Table S7). Phosphorus and calcium were also detected in surrounding tissues, albeit at much lower relative levels. To facilitate comparison, elemental ratios were used as the main quantitative parameters. Atomic ratios (Al:C, Al:P, P:C, and Al:O) showed trends similar to those of SEM-EDS; the Al:O ratio increased in both CBs and aggregates but remained below theoretical values for AlOOH and γ-Al_2_O_3_ (Figure S4). Trace elements such as N, S, K, Mg, Fe, and Cu were detected in minimal amounts and/or a majority present in control tissues (Table S7). Silicon was found in all areas but was relatively higher in CBs (Figure S5 and Table S7), which were the only ones containing low amounts of sodium (Table S7). Crystallographic analyses using fast Fourier transform (FFT) of high-resolution TEM images and selected-area electron diffraction (SAED) patterns confirmed that the crystalloid bodies (CBs) exhibited a well-ordered lattice structure, which were indexed to γ-Al_2_O_3_ (Figure 9K,L).

**Figure 9:**
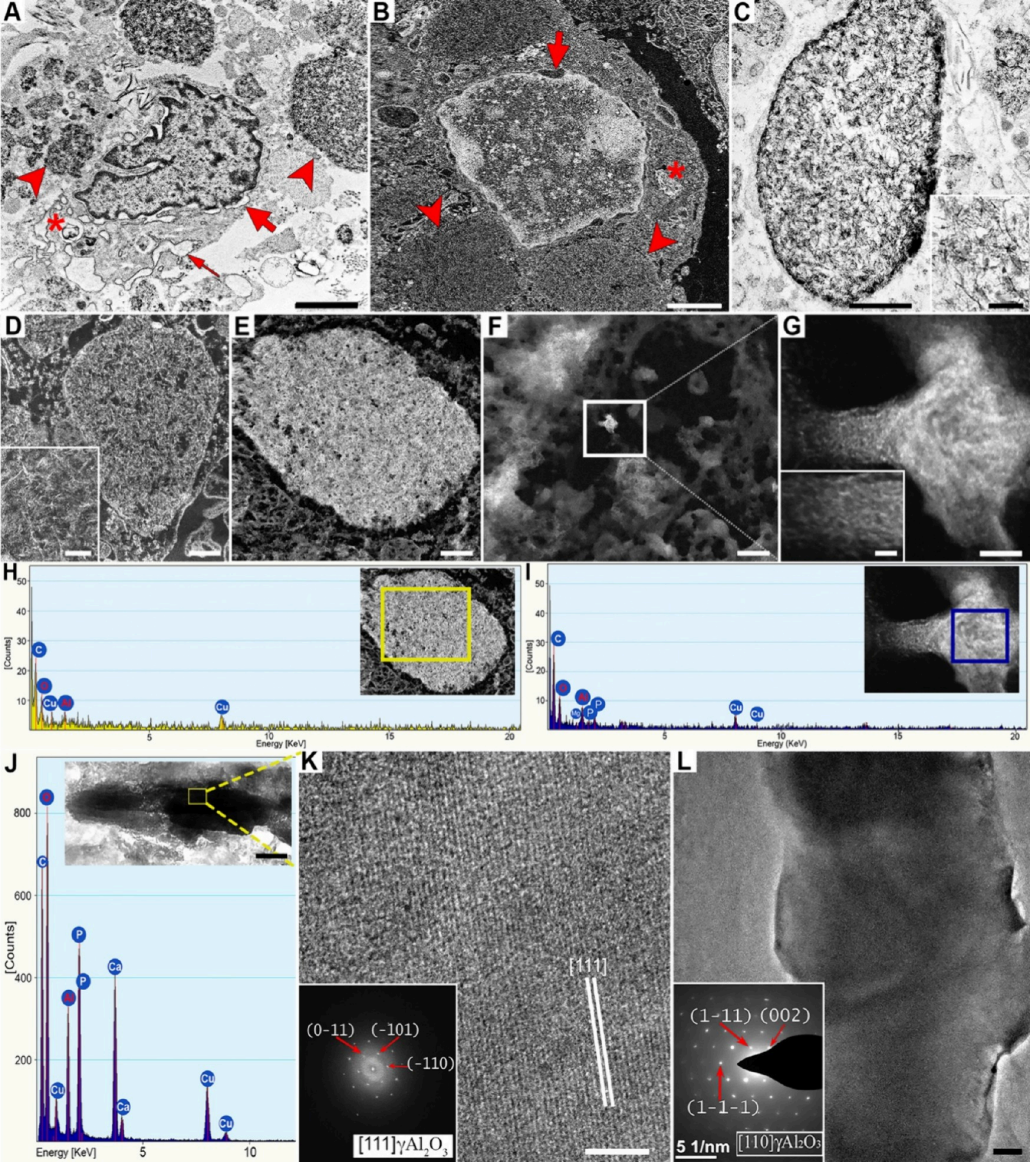
TEM and STEM analyses of subcutaneous granulomas induced by aluminum oxyhydroxide (AlOOH)-adjuvanted vaccines in sheep (ultrathin sections). (A, B) Macrophages with vacuoles filled with nanoparticles displacing nuclei (arrowheads); nuclear membrane swelling (thick arrows), abundant euchromatin/heterochromatin, swollen smooth endoplasmic reticulum (thin arrow), and frequent myelin figures (asterisks). Bars: 2 μm. (C) Membrane-bound vacuoles containing laminated, needle-like nanoparticles resembling the AlOOH adjuvant. TEM image, bar: 500 nm, inset bar: 100 nm. (D, E) HAADF-STEM image showing different aggregation stages of intravacuolar nanoparticles. Bars 500 nm; insert bar: 100 nm. (F, G) Similar nanoparticle aggregates are free in the cytosol. HAADF-STEM image; F. Bar: 500 nm; G. Bar: 20 nm; insert bar: 5 nm. (H, I) Intra- and cytoplasmic aggregates mainly composed of Al and O by EDS; some contained P, Mg was considered contamination, and Cu was derived from the grid. (J) Crystalloid bodies (CBs) visible as electron-dense structures composed of higher Al and O, with occasional P and Ca; Cu signal from the grid. Insert bar: 500 nm. (K, L) HRTEM (K, bar: 5 nm) and TEM (L, bar: 500 nm) images of CBs from two granulomas, each from a different animal included in the study (*n* = 2). Inset K shows the FFT indexed as the [111] zone axis of γ-Al_2_O_3_, where the three {110} planes of *d*_{110}_ = 0.5613 nm were observed. Inset L shows the SAED pattern of the [010] zone axis of γ-Al_2_O_3_; Spots corresponding to planes (111) and (002) *d*_(111)_ = 0.4583 nm; *d*_(002)_ = 0.3969 nm.

Other pathological changes in Al-laden macrophages included numerous concentric lamellar whorls that appeared electron dense under TEM or displayed high electron intensity under HAADF-STEM, consistent with myelin figures, as well as abundant euchromatin with peripheral heterochromatin, vesiculation of the nuclear envelope, and swelling of the endoplasmic reticulum (Figure 9A,B). However, the evaluation of further ultrastructural changes, particularly mitochondrial integrity, was unreliable due to artifacts.

Overall, intracellular Al was detected as nanoparticle aggregates within macrophages, while CBs exhibited distinct morphology, elemental composition, and well-ordered lattice structures, consistent with a crystalline phase.

### 2.4 Elemental Composition Mapping and Phase Analyses of Granulomas by Advanced SEM-EDS

A granuloma was examined using a Thermo Scientific Apreo ChemiSEM S LoVac field emission scanning electron microscope (FE-SEM) (Thermo Fisher Scientific, Eindhoven, NL). The system combines high-resolution SEM imaging with the integrated ChemiSEM platform, which provides a real-time elemental mapping. In addition, the ChemiPhase software enables automated clustering of EDS spectra through multivariate statistical analysis, allowing the identification of compositionally distinct phases based on spectral similarity.

SEM-EDS mapping on resin-embedded semithin sections confirmed the presence of Al in intracellular aggregates of Al nanoparticles both within macrophages and in the CBs, with a relatively higher weight content in CBs (Figure 10A,B). ChemiPhase clustering of SEM-EDS spectra identified three compositionally distinct phases, which corresponded morphologically to the surrounding tissue (phase 1), CBs (phase 2), and Al intracellular nanoparticles (phase 3) (Figure 10C–E). With respect to phase composition, the observed trends closely matched those obtained by SEM and TEM-EDS analyses. Phase 2 (CBs) exhibited approximately 3-fold higher atomic Al:C and P:C ratios compared with phase 3 (Al nanoparticles), which in turn showed 3-fold higher ratios than phase 1 (surrounding tissue) (Figure 10C–E). The relative enrichment of phosphorus in CBs compared with Al nanoparticles, and in nanoparticles compared with surrounding tissue, was further supported by phosphorus quant maps. Calcium was detected at measurable atomic levels exclusively in phase 2 (CBs), where it localized to crystalline structures (Figure 10D,G), whereas only trace amounts were occasionally observed in phase 3 (Al intracellular nanoparticles; Figure 10E,G). Nitrogen was variably detected across all three phases (Figure 10C–E), with mapping showing no preferential localization (Figure 10H). Complete unnormalized elemental data sets for each phase, including additional trace elements introduced during processing, are provided in Table S8.

**Figure 10:**
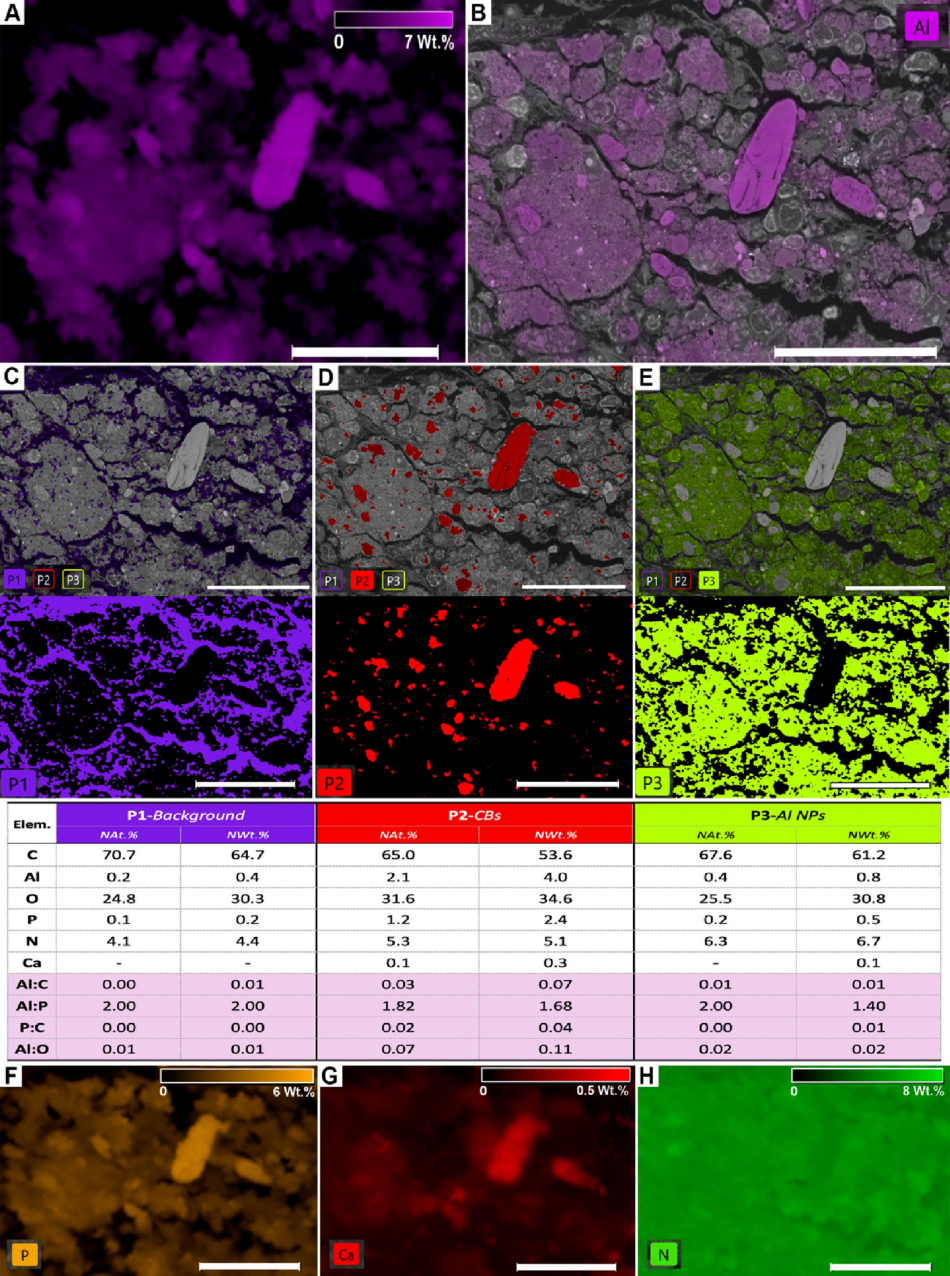
SEM-EDS elemental and phase mapping of a subcutaneous granuloma induced by an aluminum oxyhydroxide (AlOOH)-adjuvanted vaccine in sheep, performed with the ChemiSEM platform and ChemiPhase software, respectively (resin-embedded semithin sections). (A, B) Quantitative elemental maps of aluminum (Al) were obtained by SEM-EDS. (A) Al distribution expressed in weight percent (Wt%), displayed as an independent color map. (B) The same Al quant map was blended with the grayscale SEM image to illustrate its spatial relationship with tissue morphology. Scale bars: 50 μm. (C–E) ChemiPhase clustering identified three statistically distinct phases based on spectral similarity: phase 1, tissue without CBs or nanoparticles; phase 2, crystalloid bodies (CBs); phase 3, Al nanoparticles. Elemental composition of each phase is shown as normalized atomic and weight percentages (NAt.% and NWt.%), excluding elements attributable to tissue processing or staining. Ratios of Al:C, Al:P, and P:C are also provided. (F–H) Quantitative elemental maps of phosphorus (P), calcium (Ca), and nitrogen (N) obtained with ChemiSEM, expressed in Wt% and displayed as independent color maps. Scale bars: 50 μm.

Elemental mapping and phase clustering consistently identified three compositionally distinct compartments corresponding to the surrounding tissue, intracellular Al nanoparticles, and CBs, with progressive enrichment of Al and phosphorus across these phases.

### 2.5 Powder Diffraction X-ray (PXRD) Analysis of Granulomas

Transversely sectioned surfaces of formalin-fixed granulomas and a pooled sample of granulomas subjected to distilled water washings and subsequent lyophilization (see the Experimental Section) were analyzed by PXRD. The diffractograms obtained from both preparations displayed similar overall patterns (Figure S6), although the washed and lyophilized pools showed higher signal intensity and reduced background noise. The diffraction analysis was not conclusive, as only partial matching of diffraction planes corresponding to gibbsite (γ-Al­(OH)_3_), bayerite (α-Al_2_O_3_), and/or paraformaldehyde phases was observed, preventing definitive identification or exclusion of these phases (Figure S6). No γ-Al_2_O_3_ peaks were detected, and the reference γ-AlOOH (boehmite) pattern of the adjuvant exhibited considerably lower intensity than the weakest granuloma diffractogram, precluding detection or exclusion within tissue samples (Figure S7).

PXRD analyses of formalin-fixed granuloma tissue samples yielded low-intensity and partially matching diffraction patterns, limiting the definitive identification of specific Al-containing crystalline phases.

## 3 Discussion

Al-containing structures resembling microcrystals have long been observed in postvaccination granulomas induced by AlOOH in sheep.^19,26^ The term CBs was first introduced by our group in 2019.^19^ More recently, CBs have also been detected in feline injection-site sarcomas,^26^ and similar structures, though described under different names, have been reported in cats,^35^ and more rarely in other species, including humans.^28–31^ Their consistent presence within AlOOH-induced granulomas (with or without vaccine antigens)^19,32^ strongly links them to AlOOH adjuvant exposure. However, their crystalline nature, origin, and formation mechanisms remained unknown. Using samples from a previous vaccine trial in sheep,^32^ we provide the first *in situ* evidence consistent with *in vivo* crystallization of AlOOH within macrophages, with CBs identified as γ-Al_2_O_3_ crystals, establishing them as unique *in vivo* crystalline Al structures and opening new perspectives on Al-based adjuvant-cell interactions and applications.

No CB-like structures were detected in the commercial adjuvant stock (30 mg mL^–1^), in diluted adjuvant (6 mg mL^–1^) without virus, or in the vaccine administered to sheep. Only PBS-containing preparations exhibited crystalline precipitates identified by EDS and XRD as halite, consistent with dehydration artifacts from PBS during the SEM preparation. Sodium, phosphorus, and chlorine originated from the buffer, while minor elements such as sulfur or silicon likely derived from undeclared excipients of the adjuvant. In contrast, adjuvant-only samples consistently contained Al-based needle-like nanoparticles showing a broad 020 diffraction peak characteristic of pseudoboehmite, a poorly crystalline boehmite composed by crystallite of a few atomic layers,^7,36^ in agreement with previous report of the adjuvant.^13–15,17,18^ Although LDA detected micrometric nanoparticle aggregates in both adjuvant and vaccine, TEM, SEM, and XRD revealed no crystalline Al_2_O_3_ phases or CB-like structures, consistent with prior analyses of commercial AlOOH-based vaccines.^37^ Thus, the CBs found in sheep granulomas are unlikely to result from exogenous inoculation.

Having ruled out an exogenous origin, we performed *in situ* analyses using light microscopy, SEM, TEM, EDS, and crystallography to determine the composition and the *in vivo* formation of CBs. The histopathological and ultrastructural features of AlOOH-induced granulomas closely resembled those described in macrophage cultures^38^ and in injection sites of several species, including sheep, pigs, cats, and humans exposed to AlOOH adjuvants.^19,27,31,39^ Lesions consisted of globoid macrophages with eccentric nuclei and granular cytoplasm containing vacuoles filled with thin Al-based nanoparticle needles, occasionally released into the cytosol. As previously reported,^19,26^ hyaline CBs (up to 100–150 μm) were mainly intracellular, colocalized with nanoparticle aggregates, and released following macrophage necrosis. Al was consistently detected in both CBs and nanoparticles by SEM-EDS, MAH histochemistry, lumogallion fluorescence, and, for the first time, by advanced elemental mapping with Thermo Scientific Apreo ChemiSEM; no Al signal was found in macrophage nuclei.^40,41^ Crystallographic analyses based on FFTs from HRTEM images and electron diffraction patterns identified CBs as γ-Al_2_O_3_ microcrystals. Phase assignment was based primarily on crystallographic evidence from FFT and SAED, whereas EDS served as supportive compositional information rather than a definitive stoichiometric criterion. In contrast, direct characterization of AlOOH nanoparticles was not achievable due to methodological constraints described in prior *in vivo* crystallization studies.^42,43^ The ChemiPhase software of the advanced SEM-EDS consistently distinguished three phases, Al nanoparticles, CBs, and surrounding tissue, with Al:C, Al:P, and P:C ratios matching conventional SEM-EDS results, confirming their chemical distinction. CBs were enriched in Al and phosphorus relative to C, while both structures shared similar Al:P ratios, suggesting Al–P associations and supporting their origin from progressive aggregation and condensation of AlOOH nanoparticles with concurrent organic displacement. The heterogeneous Al, P, and C distributions among some CBs may represent transitional stages of this process. The lack of significant differences in Al:O ratios and their considerably lower values compared to the theoretical stoichiometries of the expected phases are understandable, given the complexity of the analyzed samples, where it is not possible to distinguish biological oxygen from that of the respective Al oxide and oxyhydroxide phases (Al_2_O_3_ and AlOOH). Additional factors such as sample oxidation or the well-documented leaching of Al from tissues fixed in buffered formalin^44^ may also contribute to these deviations. Furthermore, EDS measurements in ultrathin biological sections are influenced by factors such as section thickness and background signal, which can further affect the reliability of elemental ratios. For this reason, elemental ratios were interpreted in terms of relative associations and spatial trends (e.g., Al:P and Al:C) rather than absolute stoichiometric values. The Al:O ratio was not considered to be a reliable parameter for phase discrimination in this study. Minor elements such as silicon, occasionally observed by TEM-EDS but not confirmed by SEM-EDS, likely reflected detector-related silicon escape peaks.^45^

Crystallographic identification of γ-AlOOH nanoparticles by XRD was unfeasible due to the low signal-to-noise ratio from the surrounding organic material, the use of contrast stains (necessary for their visualization), and their inherently poor crystallinity. Additional interference from C buildup beneath the detector has been reported; moreover, unlike magnetic nanoparticles,^42^ no standardized extraction protocols exist for AlOOH nanoparticle recovery. Nonetheless, converging evidence supports their identification as AlOOH: (i) their morphology matched adjuvant particle *ex situ*-characterized preinjection; (ii) previous *in vitro* studies unequivocally identified the same nanoparticles within macrophages as AlOOH, excluding histopathological artifacts,^40^ and the resulting granulomas have been considered diagnostic of AlOOH-induced lesions,^39^ and (iii) the ChemiPhase software consistently differentiated three distinct compositionally phases, (Al nanoparticles, CBs, and surrounding tissue), through automated clustering of EDS spectra using multivariate statistical algorithms. To our knowledge, this is the first demonstration of Thermo Scientific Apreo ChemiSEM and ChemiPhase applications beyond materials science, highlighting their potential for real-time elemental mapping and automated chemical phase assignment in complex biological systems relevant to pathology, cell biology, and biomedical fields. The difference in particle thickness between the adjuvant characterized *ex situ* and the measurements obtained *in situ* may reflect the aggregation behavior of these nanoparticles, which are known to form larger, needle-like assemblies within phagolysosomes.^19,25^ Moreover, it is important to note that in this context, AlOOH adjuvant was injected together with an inactivated virus, and previous studies have shown that vaccine formulations promote even greater aggregation compared with the adjuvant alone.^19,25^

Macrophages are specialized in recognizing and internalizing foreign particulate matter. They can sequester large amounts of material within lysosomes up to an order of magnitude larger than those of most other cell types and enriched in V-ATPase proton pumps, enabling sustained lysosomal acidification.^46^ Upon contact with poorly biodegradable AlOOH nanoparticles, macrophages internalize large aggregates mainly via filopodia and macropinocytosis, independently of receptor-mediated pathways.^47^ Within few hours, the aggregates traffic to endosomal-lysosomal compartments that acidify to pH < 4–5 and occupy most of the cytoplasm.^21,25,47,48^ Some nanoparticles escape into the cytosol, triggering autophagy, but strong activation of phagocytosis suppresses autophagic clearance, leading to net retention of Al.^21^ Remarkably, macrophages tolerate this burden and remain viable for days *in vitro* and likely *in vivo*, supporting sustained nanoparticle accumulation.^25,48^

Under these conditions and given that CBs were mostly intracellular, macrophages may function as intracellular environments favoring biochemical reactions: large quantities of nanoparticles concentrate within acidic vesicles where AlOOH dissolution releases reactive Al^3+^ ions that can interact with proteins, nucleic acids, or phospholipids,^3,40,41^ potentially templating precipitation processes analogous to biomineralization.^49,50^ Supporting this interpretation, AlOOH aggregates are normally PAS- and PAS-diastase-positive,^27,39^ consistent with interactions involving polysaccharides or glycoproteins.^27^ Given the complexity of lysosomal ion homeostasis, ionic interactions may also influence these processes. The acidic pH of phagolysosomes likely promotes phosphate adsorption onto both AlOOH and γ-Al_2_O_3_ ^51^ followed by gradual calcium absorption.^52^ Whether phosphate or calcium becomes structurally incorporated into γ-Al_2_O_3_ or directly contributes to crystallization remains uncertain. Surface-bound alkyl phosphonic groups can stabilize γ-Al_2_O_3_ against rehydration to AlOOH,^53^ suggesting that phosphate may similarly enhance CB stability once formed. Macrophages, characterized by high V-ATPase expression and strong lysosomal acidification capacity, can also protonate the antibiotic clofazimine, enabling its interaction with chloride ions and subsequent formation, stabilization, and accumulation of clofazimine hydrochloride microcrystals within lysosomes.^46^ Although it involves different compounds, the methodological framework of that study, combining *in vitro* macrophage models with advanced experimental and computational approaches, could inform future investigations into the physicochemical and cellular mechanisms driving intracellular Al-based crystal formation.

To the author’s knowledge, this is the first report of true Al crystallization within an organism, specifically in mammalian cells. These observations invite a broader interpretation, where Al crystallization may be not only a cellular event but also a biologically mediated mineralization process. Biomineralization includes biologically controlled processes mediated by gene-encoded biomolecules that produce minerals for structural or functional roles (biologically induced biomineralization) or act as detoxification mechanisms (forced biomineralization).^49,50,54^ The latter has mainly been reported in microorganisms and multicellular organisms exposed to high levels of toxic metals.^54^ γ-AlOOH nanoparticles are more cytotoxic than γ-Al_2_O_3_ nanoparticles of the same characteristics due to biological reactive hydroxyl groups,^55^ and notably, many biomineralization processes occur within membrane-bound vesicles resembling phagolysosomes. CB formation may thus follow two nonexclusive possibilities: an adaptive macrophage-driven response mitigating AlOOH cytotoxicity through forced biomineralization into γ-Al_2_O_3_ or an abiotic crystallization process passively promoted by the physicochemical milieu of the phagolysosome. The former hypothesis merits further investigation, particularly in light of increasing Al exposure in humans and animals through vaccines, food, and water over recent decades.^1,3,41^

Importantly, the present study does not address the kinetics of γ-Al_2_O_3_ formation or the proportion of adjuvant undergoing transformation and therefore does not allow conclusions regarding the rate or extent of this process. In addition, while macrophages are consistently associated with these structures, the data do not demonstrate an active cellular role, and both biologically mediated and passive physicochemical processes remain plausible. These aspects warrant further investigation, as understanding the dynamics and efficiency of this transformation may be critical for elucidating the underlying mechanisms and, in the longer term, for assessing its potential relevance in biomimetic or scalable material synthesis.

A key finding of this study was the identification of CBs as genuine γ-Al_2_O_3_ crystals, with diffraction patterns matching indexed planes of this phase rather than AlOOH, consistent with an *in vivo* phase transformation. Accordingly, the term “crystalloid bodies” may be reconsidered, and descriptors, such as microcrystalline bodies or microcrystals, may more accurately reflect their nature. CBs showed perimeters consistent with the typical growth habits of γ-Al_2_O_3_, while their elongated, needle-like morphologies likely represent differently oriented sections of CBs, possibly corresponding to thin γ-Al_2_O_3_ flakes, as described.^56^ This may also explain the difficulty in obtaining complete ultrathin sections of CBs in TEM or HAADF-STEM without displacement, further compounded by the limited area examined, which precluded a detailed characterization of the surrounding ultrastructural context. Similar limitations have also been noted in other *in vivo* protein crystallization studies.^43^ Overall observations suggest that the dehydration process usually required to transform AlOOH into γ-Al_2_O_3_, normally achieved by calcination above 450 °C and prolonged reaction times,^9,56,57^ may occur in aqueous, low-temperature biological environments. From a materials science perspective, this represents a key innovative aspect of the study as it points to a fundamentally different route for γ-Al_2_O_3_ formation that could reduce energy requirements and processing constraints. Such transformation under soft conditions parallels biomineralization processes that have inspired biomimetic synthesis of crystalline materials through greener routes.^49,50,54^ Given the well-established industrial value of γ-Al_2_O_3_, including applications in catalysis, optoelectronics, ceramics, adsorption technologies, bioremediation, and biomedical fields such as drug delivery,^9,11,56,58–60^ these findings provide conceptual inspiration for more sustainable and energy-efficient synthesis strategies. The association of macrophages with γ-Al_2_O_3_ formation through unconventional biological pathways further suggests that cells may act as intracellular environments, enabling such transformations under mild conditions. Future investigations into the biological and physicochemical mechanisms underlying this process, including potential self-assembly phenomena, the role of calcium and phosphate ions at the organic–inorganic interface, and the specific conditions of the phagolysosomal environment,^46^ may help to determine whether these processes can be understood and eventually controlled. In this context and if these mechanisms are elucidated, such transformations could be framed within emerging materials science concepts such as nanoarchitectonics,^61^ where controlled assembly at the nanoscale enables the design of functional materials, potentially opening new avenues for biomimetic γ-Al_2_O_3_ synthesis under mild and energy-efficient and green conditions.

Similar crystalloid body-like structures have been described in other species,^19,20,26,27^ including humans,^28–31^ suggesting that this phenomenon may extend beyond the present experimental model. However, its extent and biological relevance across species remain to be determined. Future studies incorporating multiple time points will be necessary to better understand the kinetics and progression of γ-Al_2_O_3_ crystal formation, as previous longitudinal studies of ovine granulomas have not specifically addressed the evaluation of these crystalline structures.^20^ In addition, controlled comparisons including adjuvant-only conditions and biodistribution analyses across different tissues may help clarify the factors influencing crystal formation and potential systemic implications.

Beyond its structural significance, the occurrence of γ-Al_2_O_3_ crystals within macrophages raises biological questions regarding adjuvant persistence, cellular stress responses, and systemic migration. The presence of γ-Al_2_O_3_ crystals may have implications for vaccine-induced immunity, pathology, and adjuvant toxicology. A key concern regarding AlOOH nanoparticles is their potential migration to distant organs, including the nervous system, where toxicity has been suggested.^21,25,48,62^ In the same vaccinated sheep, a previous study found a positive correlation between the amount of inactivated virus and macrophage migration carrying Al nanoparticles to lymph nodes, which was inversely related to the number and size of CBs at injection sites.^32^ This indicates that macrophages and local AlOOH availability are central to CB formation: greater migration reduces the local material for crystallization, while limited migration favors accumulation and crystal growth. If confirmed, CBs could serve as indicators of the AlOOH nanoparticle biodistribution, providing a valuable framework for future toxicological research.

Sample preparation artifacts should be considered when interpreting these structures. However, several observations argue against their origin being solely induced *ex vivo* by fixation, embedding, or sectioning. Similar CBs have been consistently reported across different studies, commercial vaccine formulations, adjuvant loads, and varying postvaccination and sampling time points, as well as after distinct tissue-processing workflows and fixation methods, including nonparaffin-embedded material.^19,20^ Notably, comparable crystalline structures were observed using both Tokuyasu cryo-sectioning and conventional resin embedding approaches, which rely on fundamentally different preparation workflows and associated artifacts. The consistent detection of these structures across methods supports the idea that their presence is not attributable to a single preparation technique. Nevertheless, preparation-related effects cannot be fully excluded, and further studies using freshly collected, unfixed tissue in combination with advanced cryo-preservation approaches may help clarify their origin. However, the potential effects of freezing on the aggregation state of AlOOH nanoparticles should also be considered in future cryo-preservation-based studies.^63^

The inability of the PXRD analyses of the granulomas to detect γ-Al_2_O_3_ likely reflects the very low abundance of these crystalline structures relative to the surrounding organic matrix, a recognized limitation in similar *in cellulo* crystallization studies of proteins.^43^ Moreover, the large proportion of organic material relative to inorganic crystalline content in these samples further complicates the accurate identification of inorganic phases by attenuating the overall diffraction signal intensity.^43^ Consequently, even though γ-AlOOH nanoparticles or γ-Al_2_O_3_ microcrystals were microscopically visualized, the occurrence of boehmite or other aluminum hydroxide phases within the granulomas, such as gibbsite or bayerite, could not be entirely ruled out and warrants further investigation.

## 4 Conclusions

This study provides evidence consistent with Al crystallization within mammalian cells and identifies macrophages in vaccine-adjuvant-induced granulomas as potential biological environments associated with the formation of γ-Al_2_O_3_ microcrystals from γ-AlOOH (pseudoboehmite). The observation that γ-Al_2_O_3_ may form under mild, aqueous, and biologically relevant conditions challenges conventional assumptions regarding its formation, which typically rely on high-temperature calcination and may inspire future low-energy, environmentally friendly biomimetic approaches to its synthesis. Such approaches, if further understood, including the elucidation of potential underlying biological self-assembly processes, could be relevant for energy-efficient and sustainable applications in the materials industry, including catalysis, adsorption technologies, and bioremediation, as well as for biomedical applications such as drug delivery. In this context, these findings may also be conceptually related to emerging materials science frameworks such as nanoarchitectonics, in which functional materials arise from hierarchical and dynamic assembly processes. However, further mechanistic studies are required to determine whether such concepts can be meaningfully applied in this system. Beyond its materials science implications, this finding also raises biological questions regarding potential cellular adaptations of macrophages to persistent aluminum exposure, suggesting that these cells may contribute, through active or passive processes, to modulating the physicochemical state of the adjuvant within the tissue environment. As this crystal formation appears to be associated with vaccine formulations and related factors, it may also have relevance from an immunotoxicological and pathological perspective. In addition, the use of EDS-based compositional mapping techniques in biological tissues, commonly employed in mineralogy, may offer new opportunities for the *in situ* identification of exogenous materials within lesions, with a potential relevance for diagnostic and forensic pathology.

## 5 Experimental Section

### Animals and Vaccine Formulation

The experimental design and all procedures were approved by the Ethics Committee of the University of Zaragoza (PI34–18) and authorized by the regional competent authority (Dirección General de Alimentación y Fomento Agroalimentario, Gobierno de Aragón), in accordance with Spanish legislation (Royal Decree 53/2013), which implements Directive 2010/63/EU of the European Parliament for the protection of animals used for scientific purposes. As part of a previously published experiment,^32^ 30 13-month-old Rasa Aragonesa male sheep (50.0 ± 6.8 kg) were sourced from a certified disease-free flock; all had been surgically castrated prior to the study. From this cohort, six animals (53.5 ± 6.8 kg) received an inactivated bluetongue virus serotype 4 (BTV-4) vaccine containing strain BTV-4/SPA-1/2004 (CZ Vaccines, Porriño, Spain). The antigen was formulated in PBS and adjuvanted with 6 mg mL^–1^ AlOOH (Adjuval, CZ Vaccines). Each dose consisted of 2 mL, providing 12 mg of AlOOH (0.23 ± 0.03 mg kg^–1^), equivalent to approximately 4.68 mg of Al^3+^, as determined by triplicate ICP-AES analysis of PBS preparations containing 6 mg mL^–1^ of AlOOH (2.34 ± 0.05 mg of Al^3+^). A control group of six sheep was vaccinated with the same BTV-4 antigen formulated in PBS without AlOOH, following the same administration protocol.

### *Ex Situ* Characterization of AlOOH Adjuvant and Its Formulated Vaccine

The AlOOH adjuvant (Adjuval, CZ Vaccines) and the formulated vaccine were characterized *ex situ* using complementary structural and morphological techniques. Powder X-ray diffraction (PXRD) was performed on dried samples using a PANalytical Empyrean diffractometer (Cu Kα radiation, λ = 1.5406 Å, 45 kV, 40 mA) in Bragg–Brentano geometry. Diffractograms were collected over 2θ = 10–90° with a 0.01° step size during 1 h and processed using OriginPro 9.8 (OriginLab Corporation, USA). Particle morphology and aggregation state were evaluated by transmission electron microscopy (TEM) and high-angle annular dark-field scanning TEM (HAADF-STEM). For these assays, 0.1 mg mL^–1^ suspensions in PBS were deposited onto carbon-coated copper grids and imaged using FEI Tecnai instruments operated at 200–300 kV. To screen for the presence of CBs in the adjuvant stock and vaccine formulations, both undiluted samples (30 mg mL^–1^) and dilutions matching the vaccine concentration (6 mg mL^–1^) were examined by scanning electron microscopy (SEM) using a Thermo Fisher Scientific Inspect F50 microscope at 10 kV. Same vaccines used for immunization were analyzed undiluted and at 1:5 and 1:10 dilutions following carbon coating. Aggregate size distribution in PBS and within the vaccine matrix was quantified by laser diffraction (Mastersizer 3000E, Malvern Instruments, UK) by using a HydroSV dispersion unit. Measurements were performed in triplicate, and volume-based parameters (D­[4,3], Dv10, Dv50, Dv90, span) were calculated using Mie theory.

### *In Vivo* Experimental Schedule

Animals were vaccinated subcutaneously in the dorsal scapular region with the first dose administered on the right side and the second on the left, 21 days apart. Injection sites were shaved before each administration to ensure a consistent localization. All animals were humanely euthanized 133 days after the first vaccination by intravenous injection of pentobarbital (Dolethal).

### Post-mortem Sampling and Histopathological Analysis

Injection sites were identified and inspected in all animals prior to sampling. Subcutaneous tissues were exposed, dissected, and examined for inflammatory changes following previously described procedures.^19^ All injection sites from the AlOOH-vaccinated (*n* = 12) and virus-only (*n* = 12) groups were collected for histopathology. Tissues were fixed in 10% neutral-buffered formalin, paraffin-embedded, sectioned at 4 μm, and stained with hematoxylin and eosin (H&E), modified Al-hematoxylin (MAH), and lumogallion fluorescence following established protocols.^19,26,64–66^ For lumogallion staining, a consecutive buffer-only section from each sample was evaluated to rule out tissue autofluorescence (negative controls). Aluminum-based CBs were assessed in H&E-stained sections for morphology, length, width, and aspect ratio at 20× magnification using ImageJ.

### Electron Microscopy Sample Preparation and Analysis

Formalin-fixed granuloma samples (*n* = 4) were reprocessed for electron microscopy using standard glutaraldehyde fixation and buffer-based washing procedures. Ultrathin sections were generated by Tokuyasu cryo-sectioning and epoxy–resin embedding, with or without heavy-metal contrast, following established protocols. Complete processing details are provided in Supporting Information Text 1.

Transmission electron microscopy (TEM), selected-area electron diffraction (SAED), fast Fourier transform (FFT), and high-angle annular dark-field scanning TEM (HAADF-STEM) were used to assess the morphology, crystallinity, and elemental composition of adjuvant nanoparticles of AlOOH and Al-based CBs. Elemental analysis was performed on unstained and stained sections using energy-dispersive X-ray spectroscopy (EDS) equipped with an EDAX detector. Imaging was performed on a Tecnai F30 microscope operating at 300 kV and TEM images were acquired with a Gatan CCD camera.

EDS data were processed by evaluating both non-normalized and normalized elemental compositions. Normalized values were obtained after excluding trace elements present at low or inconsistent levels as well as signals attributable to staining, sample preparation, contamination (e.g., uranyl, vanadium, osmium, iron), and supporting materials (e.g., copper, chlorine). All detected elements, including those excluded from normalization, are reported in the Supporting Information to ensure transparency and allow direct comparison between normalized and non-normalized data sets. Elemental compositions were interpreted in terms of relative distributions and associations rather than absolute quantitative values. Analyses were performed across multiple regions of interest to assess the consistency of the elemental distributions within the samples.

HAADF-STEM images were obtained with a HAADF detector (Fichione); in this mode, signal intensity is proportional to the square of the atomic number (*Z* ^2^), allowing heavy elements to be visualized with higher contrast than lighter ones such as carbon or silicon. This is particularly useful for localizing metals within organic matrices.

### SEM and SEM-EDS in Tissues

Semithin resin sections (∼2 μm, see Supporting Information Text 1) and the surface of stained resin blocks were examined by scanning electron microscopy (SEM). Sections were performed using a diamond knife on a Leica EM UC7-FC7 ultramicrotome, collected on glow-discharged carbon tape mounted on aluminum stubs, coated with 20 nm of carbon, and imaged using an Inspect F50 SEM (Thermo Fisher Scientific) operating at 10 kV with secondary-electron (ETD) and backscattered-electron (BSED) detectors. Point elemental analyses were performed by using the integrated EDAX EDS system. Additionally, semithin sections from one of these granulomas were examined by using a Thermo Scientific Apreo ChemiSEM S LoVac field emission scanning electron microscope (FE-SEM) (Thermo Fisher Scientific, Eindhoven, NL). The microscope was equipped with the Trinity Detection System (in-lens SE/BSE detectors) and a TrueSight energy-dispersive X-ray spectroscopy (EDS) detector. Elemental analysis and semiquantitative chemical mapping were performed using the integrated ChemiSEM platform. The system combines high-resolution SEM imaging with the integrated ChemiSEM platform, which provides real-time elemental mapping. In addition, the ChemiPhase software enables automated clustering of EDS spectra through multivariate statistical analysis, allowing identification of compositionally distinct phases based on spectral similarity.

SEM images were digitally colorized postacquisition (Adobe Photoshop 2025) solely to aid visualization; color overlays do not represent elemental distribution. Original grayscale images are available upon request.

### PXRD of Granulomas

To determine the presence of γ-Al_2_O_3_ or other aluminum oxide/oxyhydroxide phases, two formalin-fixed granulomas were sectioned into several millimeter slices and analyzed by powder X-ray diffraction (PXRD) using a PANalytical Empyrean diffractometer in Bragg–Brentano geometry (Cu Kα, λ = 1.54 Å; 45 kV, 40 mA). Diffractograms were acquired over 2θ = 10°–90° with a 0.01° step size and 30–60 min acquisition time. To increase sensitivity for low-abundance γ-Al_2_O_3_, small fragments (1–2 mm^3^) from four additional granulomas were pooled, washed, frozen at −80 °C, lyophilized, and subjected to the same PXRD analysis

### Statistical Analysis

All statistical analyses were conducted in IBM SPSS Statistics v19.0 (IBM Corp., Armonk, NY, USA); figures were generated with OriginPro v9.8 (OriginLab, Northampton, MA, USA). Data normality was assessed using the Shapiro–Wilk test. Ratios of Al:C, Al:P, and P:C in Al nanoparticle measurements, CBs measurements, and surrounding tissue measurements were compared using the Kruskal–Wallis test with Mann–Whitney U tests for post hoc pairwise comparisons. For CBs with high relative calcium quantities and intensities, versus the remaining CBs, either Student’s *t* test or Mann–Whitney *U* test was applied, depending on normality. Correlations between ratios were evaluated with Spearman’s rho tests, as appropriate. Statistical significance was set at *p* < 0.05.

## Supplementary Material



## Data Availability

The data that support the findings of this study are available within the article and its Supporting Information.

## Notes

Tissue processing protocol for electron microscopy; hypertrophic capillaries, fibroblasts, and neutrophils identified by STEM analysis in subcutaneous aluminum oxyhydroxide (AlOOH)-granulomas of sheep; intramacrophage cytosolic aggregates of AlOOH adjuvant analyzed by STEM-HAADF and EDS, voids at the place of crystalloid bodies (CBs) within AlOOH-granulomas in sheep analyzed by STEM; atomic ratios obtained from TEM-EDS analyses of CBs, aluminum aggregates (Al Aggr), and surrounding tissue in subcutaneous granulomas induced by AlOOH-adjuvanted vaccines in sheep; EDS analysis of a CB showing high relative quantities of silicon (Si) in addition to oxygen (O) and aluminum (Al) in unstained ultrathin sections; PXRD patterns of formalin-fixed granuloma sections (cut surface, no washings) and a pooled sample of washed and lyophilized granulomas; PXRD diffractograms of surface sections from two formalin-fixed granulomas in sheep (Tissues); Table data of laser diffraction analysis of aluminum oxyhydroxide (AlOOH) adjuvant nanoparticles alone and within the vaccine; table data of SEM-EDS analysis with unnormalized elemental composition of CBs, Al Aggr, and surrounding tissues; statistical comparison of elemental atomic percentage (at. %) ratios from SEM-EDS analyses of CBs, Al aggr., and surrounding tissue; statistical correlation of elemental atomic percentage (at. %) ratios from SEM-EDS analyses of CBs and intracytoplasmic Al aggr.; statistical comparison of elemental atomic (at.%) ratios between CBs with high electron signal intensity and detectable calcium (Ca), and CBs with low electron intensity and no detectable Ca; statistical correlation of elemental atomic (at. %) ratios between CBs with high electron signal intensity and detectable calcium (Ca), and CBs with low electron intensity signals and no detectable Ca; table data of SEM-EDS analysis with complete unnormalized elemental composition of CBs, Al Aggr, and surrounding tissues; breakdown of all elements detected in background tissue (phase 1), crystalloid bodies (CBs, phase 2), and aluminum nanoparticles (Al NPs, phase 3) identified by SEM-ChemiPhase analysis (PDF)

## †

Echevarne Laboratory, 312 de Provença Street, Eixample, Barcelona 08037, Spain

## ABBREVIATIONS

Al: aluminum
O: oxygen
AlOOH: aluminum oxyhydroxide
CBs: crystalloid bodies
PBS: phosphate buffered saline
TEM: transmission electron microscopy
HAADF-STEM: transmission electron microscopy
LDA: laser diffraction analysis
PXRD: powder X-ray diffraction
SEM: scanning electron microscopy
MAH: modified aluminum-hematoxylin
AR: aspect ratio
BSED: backscattered electron detector
ETD: Everhart–Thornley detector
EDS: energy-dispersive spectroscopy
HRTEM: high-resolution transmission electron microscopy
FFT: fast Fourier transform
