# Supplementary material for: Macrophage-Mediated In Vivo Intracellular Crystallization of Aluminum Oxyhydroxide Adjuvant in Vaccine-Induced Granulomas
Source: ACS Appl Mater Interfaces. 2026 Apr 30;18(18):25960–78. doi: 10.1021/acsami.6c04089 (PMC13181710; doi:10.1021/acsami.6c04089)
Supplement: Supplementary file 1 [file am6c04089_si_001.pdf]

Supporting information

Macrophage-mediated in vivo intracellular crystallization of aluminum oxyhydroxide adjuvant in vaccine-induced granulomas

*Estela Pérez[a]†, Marta Navarro[bc], Alfonso Ibarra[bc], Ignacio de Blas[d], Marta Pérez[ed], Lluís Luján[ad], Víctor Sebastián[bcfg]**

[a] Department of Animal Pathology, University of Zaragoza, 177 Miguel Servet Street, Zaragoza, 50013, Spain.

[b]Institute of Nanoscience and Materials of Aragon (INMA), CSIC-University of Zaragoza, Mariano Esquillor Gómez Street I+D+i building, Zaragoza, 50018, Spain.

[c]Advanced Microscopy Laboratory, University of Zaragoza, Mariano Esquillor Gómez Street I+D+i building, Zaragoza, 50018, Spain.

[d]Agri-Food Institute of Aragon (IA2), University of Zaragoza, 177 Miguel Servet Street, Zaragoza, 50013, Spain.

[e]Department of Anatomy, Embryology and Genetics, University of Zaragoza, 177 Miguel Servet Street, 50013, Spain.

[f] Department of Chemical and Environmental Engineering, University of Zaragoza, 3 María de Luna Street, Zaragoza, 50018, Spain.

[g] Networking Research Center on Bioengineering, Biomaterials and Nanomedicine (CIBER-BBN), 3-4 Monforte de Lemos Street, Madrid, 28029, Spain.

*E-mail: victorse@unizar.es

Supporting information index

1. Supporting Text ... ***3***
Text S1. ... 3
2. Supporting Figures ... ***5***
Figure S1 ... 5
Figure S2 ... 6
Figure S3 ... 7
Figure S4. ... 8
Figure S5 ... 9
Figure S6. ... 10
Figure S7. ... 11
3. Supporting Tables ... ***12***
Table S1... 12
Table S2... 13
Table S3... 14
Table S4... 15
Table S5... 16
Table S6... 17
Table S7... 18
Table S8... 19

1. Supporting Text

Text S1. Tissue processing for electron microscopy

The selected granuloma samples were initially fixed in 10% neutral-buffered formalin. Thus, tissue processing began with the recovery of these formalin-fixed samples by re-fixation in a glutaraldehyde-based fixative. For this purpose, 1 mm^3 fragments from each granuloma ($n = 4$) were rinsed in 0.1 M phosphate-buffered saline (PBS) and subsequently immersed in 2.5 % glutaraldehyde prepared in 0.1 M phosphate buffer (PB) for 2 hours at room temperature (20-22 °C). The tissue fragments were then washed three times for 2 minutes each in 0.1 M PB (pH 7.4) at room temperature and stored at 4 °C in the same buffer until further processing. To enable proper sectioning of crystalloid bodies for both visualization and chemical analysis, ultrathin sections were prepared using two protocols: Tokuyasu cryo-sectioning and resin embedding, with and without contrast agents. For Tokuyasu cryo-sectioning, samples were washed three times in 0.1 M PBS (5 min each) and once in 0.15 % glycine in PBS (10 min), followed by sequential incubations at 37 °C in 1 % and 12 % gelatin in 0.1 M PBS for 10 and 15 minutes, respectively. The gelatin-embedded blocks were solidified on ice at 4 °C for 30 minutes and then infiltrated overnight at 4 °C with 2.3 M sucrose on a rotor. Ultrathin sections (<70 nm) were cut at -110 °C using a Leica EM UC7-FC7 cryo-ultramicrotome equipped with a DiATOME cryo-immune diamond knife. Sections were collected in a 1:1 mixture of 2.3 M sucrose and 0.1 M PBS with 2 % methylcellulose, transferred to carbon/Formvar-coated 100-mesh hexagonal copper grids, and stored at 4 °C. Prior to TEM imaging, grids were rinsed in PBS for 5 min, followed by five consecutive 1-min washes in distilled water to remove residual sucrose and gelatin. Sections were either imaged directly or stained beforehand. Ultrathin sections were stained with 0.6 % uranyl acetate in distilled water and 0.2 % methyl cellulose in distilled water for 1 h at room temperature, then washed with distilled water. Samples were also embedded in resin, sectioned at room temperature, and either subjected to heavy metal staining or left unstained with osmium tetroxide, uranyl acetate, and lead citrate. For this, tissue fragments (~1 mm^3) were fixed overnight at room temperature in 2.5 % glutaraldehyde and 2 % paraformaldehyde. After rinsing in 0.1 M PBS (2 × 5 min), samples were embedded in agarose blocks and post-fixed in 1 % osmium tetroxide and 1.5 % potassium ferricyanide at 4 °C for 2 h. *En bloc* staining was performed using 0.5 % uranyl acetate in distilled water at 4 °C for 1 h. Dehydration was carried out in graded acetone: 70 % (overnight at 4 °C), 90 % (1 × 15 min), 96 % (1 × 15 min), and 100 % (3 × 30 min). Samples were then infiltrated with EMbed 812 resin mixed with acetone (ratios of 3:1 and 1:1 for 30 min each; 1:3 for 45 min at 37 °C), followed by pure resin (30 min at 37 °C). Polymerization was completed at 60 °C for 72 h. Ultrathin sections (<70 nm) were cut using

a diamond knife and mounted on carbon/Formvar-coated 100-mesh hexagonal copper grids. Ultrathin sections were further contrasted with 2 % uranyl acetate and 3 % lead citrate.

2. Supporting Figures

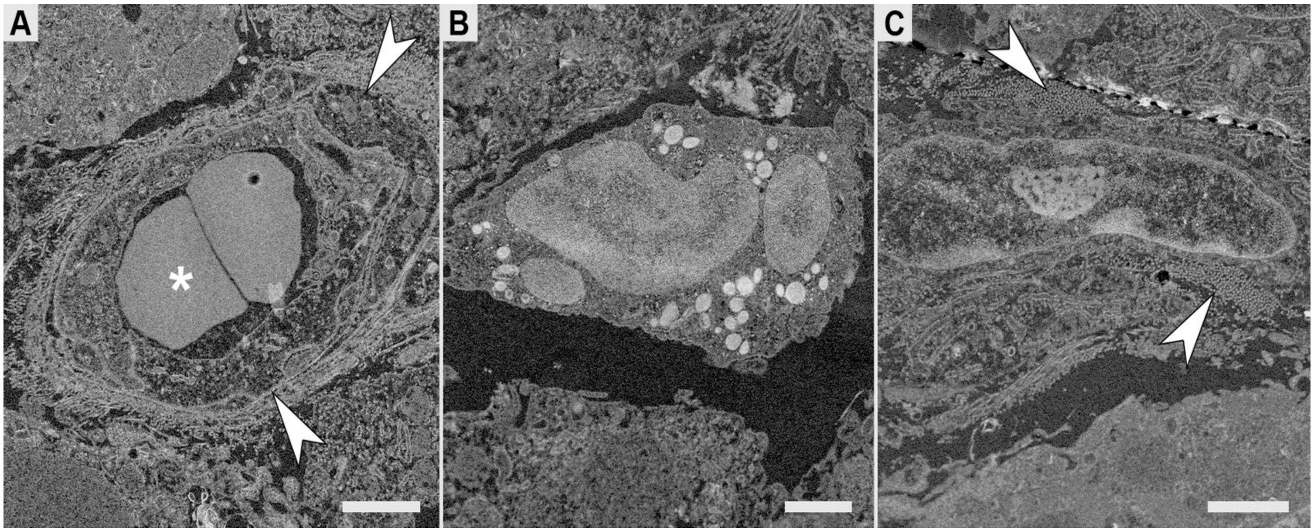

Figure S1. STEM analysis of subcutaneous granulomas induced by aluminum oxyhydroxide (AlOOH)-adjuvanted vaccines in sheep (resin-embedded ultrathin sections). **A.** Capillary lined by hypertrophic, reactive endothelium (arrowheads), containing intravascular red blood cells (asterisk). Bar = 2 µm. **B.** Neutrophil. Bar = 1 µm. **C.** Fibroblast with active nucleus surrounded by bundles of collagen fibers (arrowheads). Bar = 2 µm.

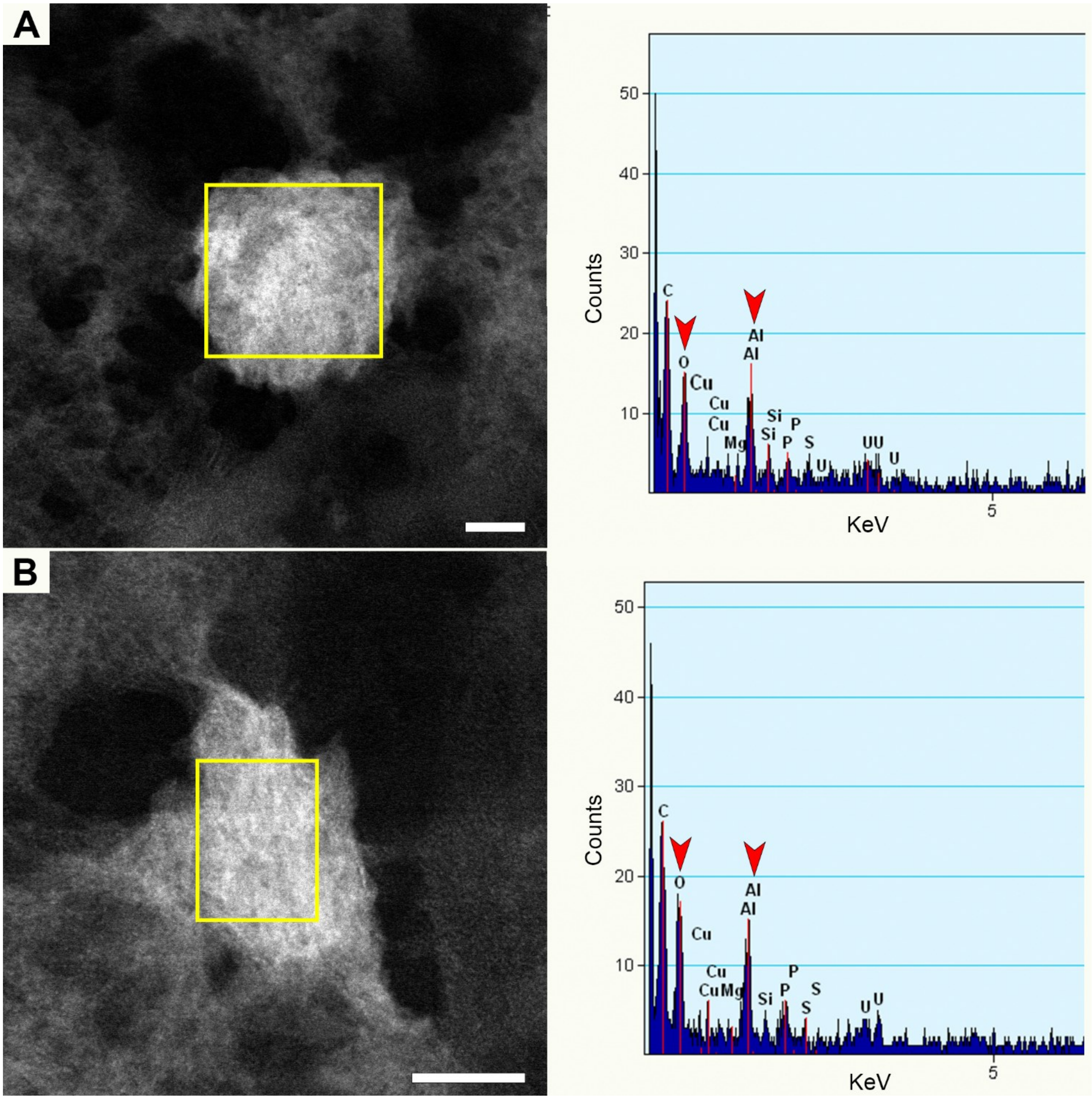

Figure S2. STEM-HAADF and EDS analyses of subcutaneous granulomas induced by aluminum oxyhydroxide (AlOOH)-adjuvanted vaccines in sheep (stained ultrathin cryosections). Cytosolic aggregates of adjuvant nanoparticles showed oxygen (O), aluminum (Al) (arrowheads), and phosphorus (P) peaks. Additional signals of silicon (Si) and sulfur (S) were detected at lower relative levels. Magnesium (Mg) was also detected at low levels. Copper (Cu) and uranium (U) signals originated from the grid and electron microscopy staining, respectively. Bars = 50 nm.

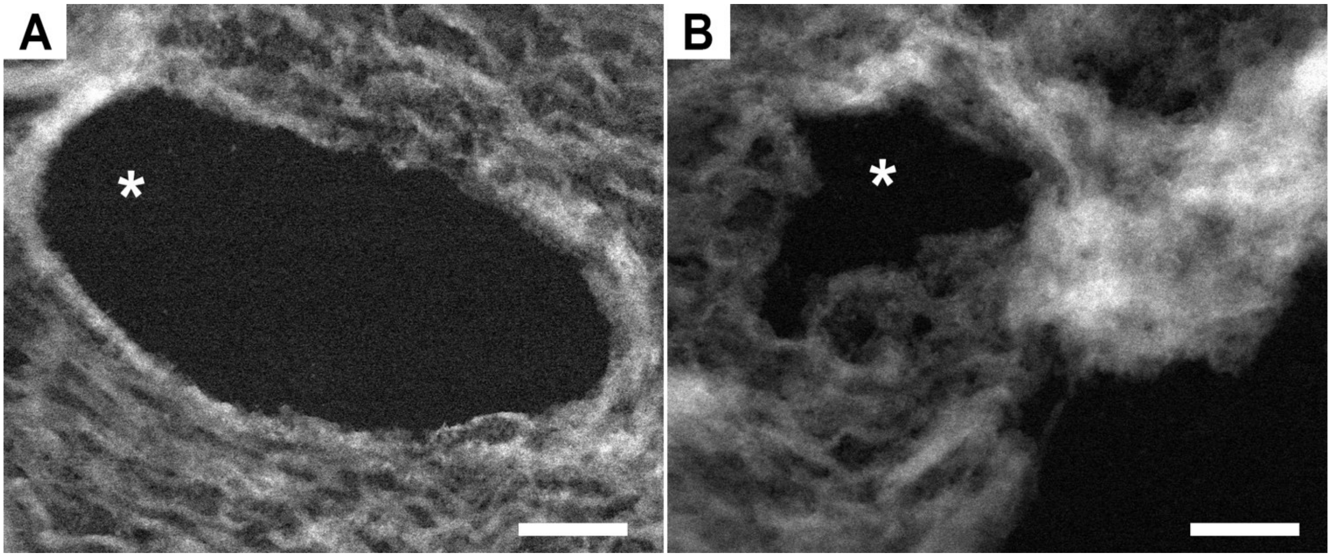

Figure S3. STEM analyses of subcutaneous granulomas induced by aluminum oxyhydroxide (AlOOH)-adjuvanted vaccines in sheep (unstained ultrathin cryosections). **A-B.** Electron signal voids (asterisks) were frequently observed. Some exhibited morphologies consistent with crystalloid bodies (CBs), as identified by complementary techniques such as light microscopy and SEM. Bars = 2 µm.

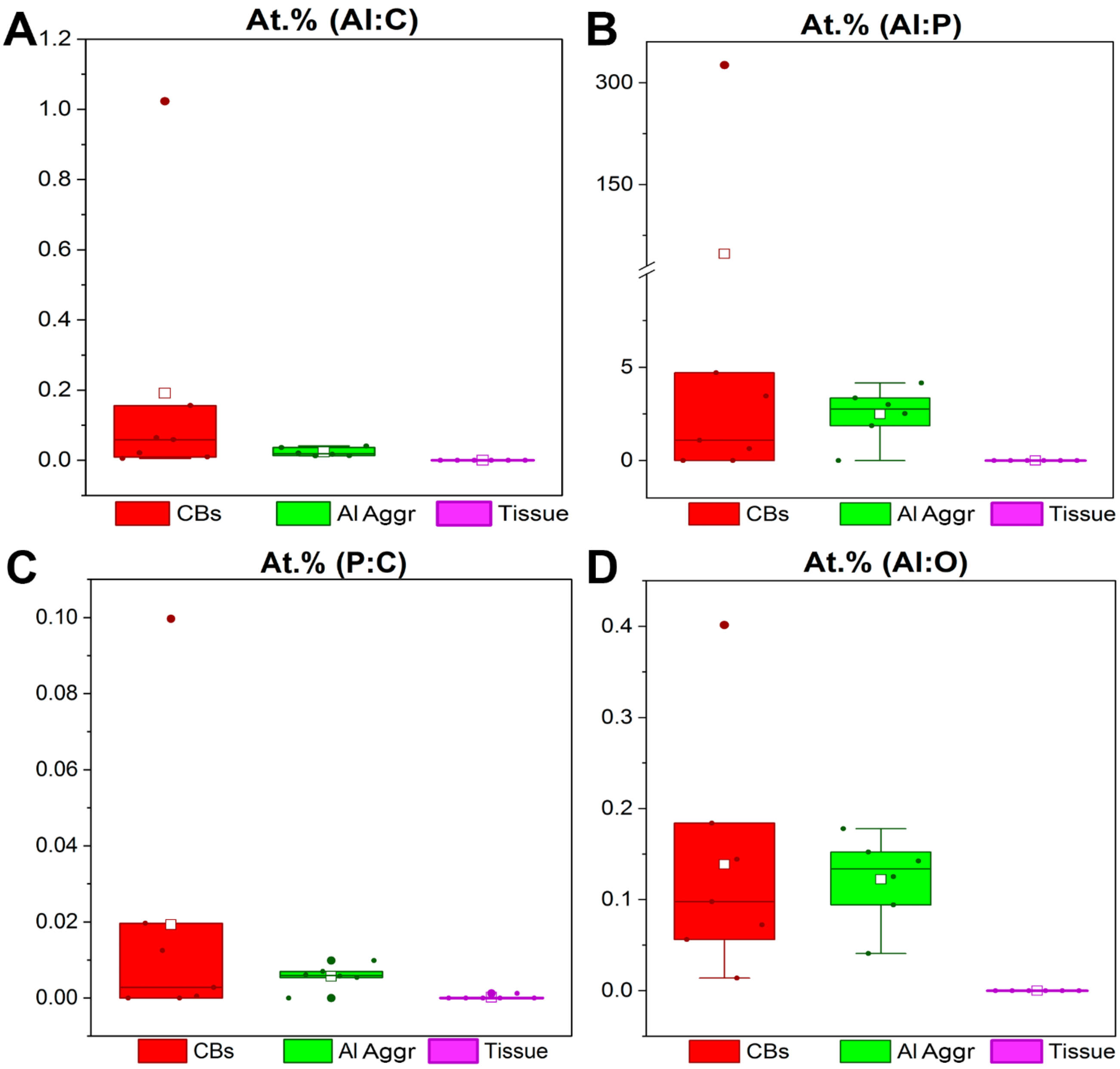

Figure S4. Atomic ratios obtained from TEM-EDS analyses of crystalloid bodies (CBs), aluminum aggregates (Al Aggr.), and surrounding tissue in subcutaneous granulomas induced by aluminum oxyhydroxide (AlOOH)-adjuvanted vaccines in sheep. **A.** aluminum to carbon (Al:C); **B.** aluminum to phosphorus (Al:P); **C.** phosphorus to carbon (P:C); **D**. aluminum to oxygen (Al:O) ratios. Boxplots show interquartile ranges with medians (horizontal lines), means (white squares), and whiskers representing minimum and maximum values; outliers are shown as larger dots.

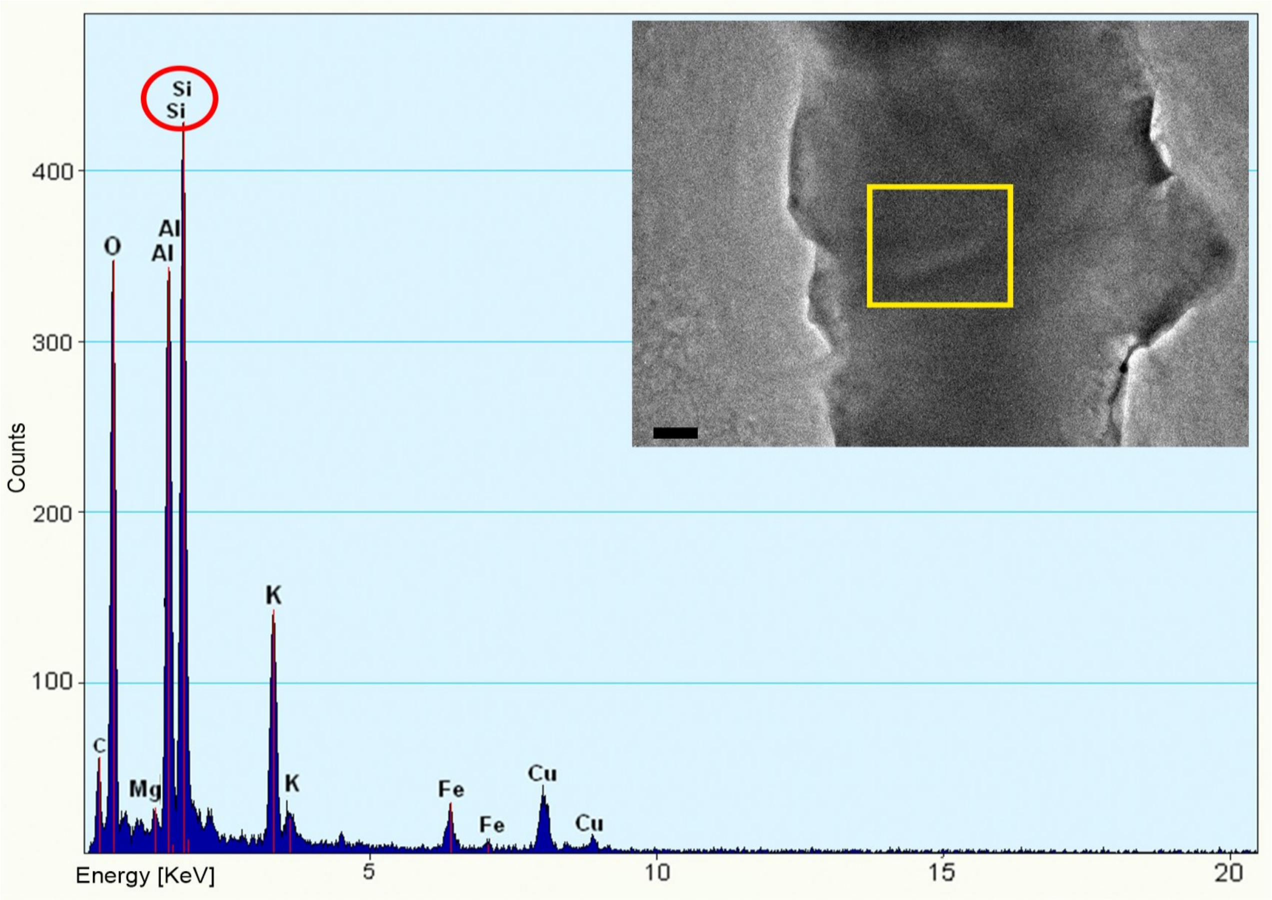

Figure S5. EDS analysis of a crystalloid body (CB) showed relatively high levels of silicon (Si) along with oxygen (O) and aluminum (Al) in unstained ultrathin sections. Trace amounts of magnesium (Mg) were also detected. Potassium (K) and iron (Fe) were attributed to electron microscopy processing, and copper (Cu) to the grid. Insert bar = 0.5 µm.

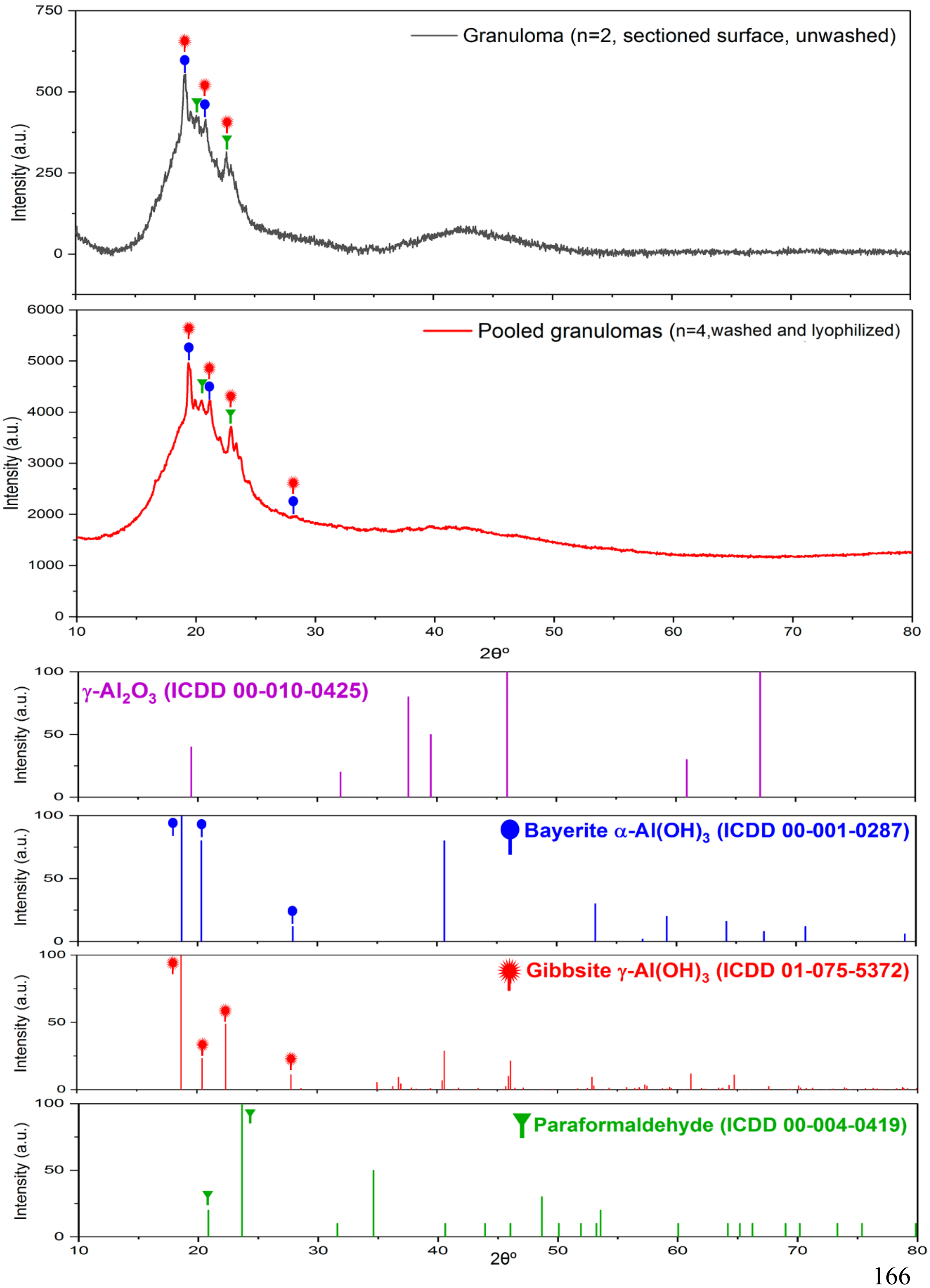

Figure S6. PXRD patterns of formalin-fixed granuloma sections (cut surface, no washings) and a pooled sample of washed and lyophilized granulomas. Reference diffraction planes for alumina (γ-Al_2O_3), gibbsite (γ-$Al(OH)_3$), bayerite (α-$Al(OH)_3$) and paraformaldehyde are shown for comparison. Corresponding diffraction planes are marked by symbols.

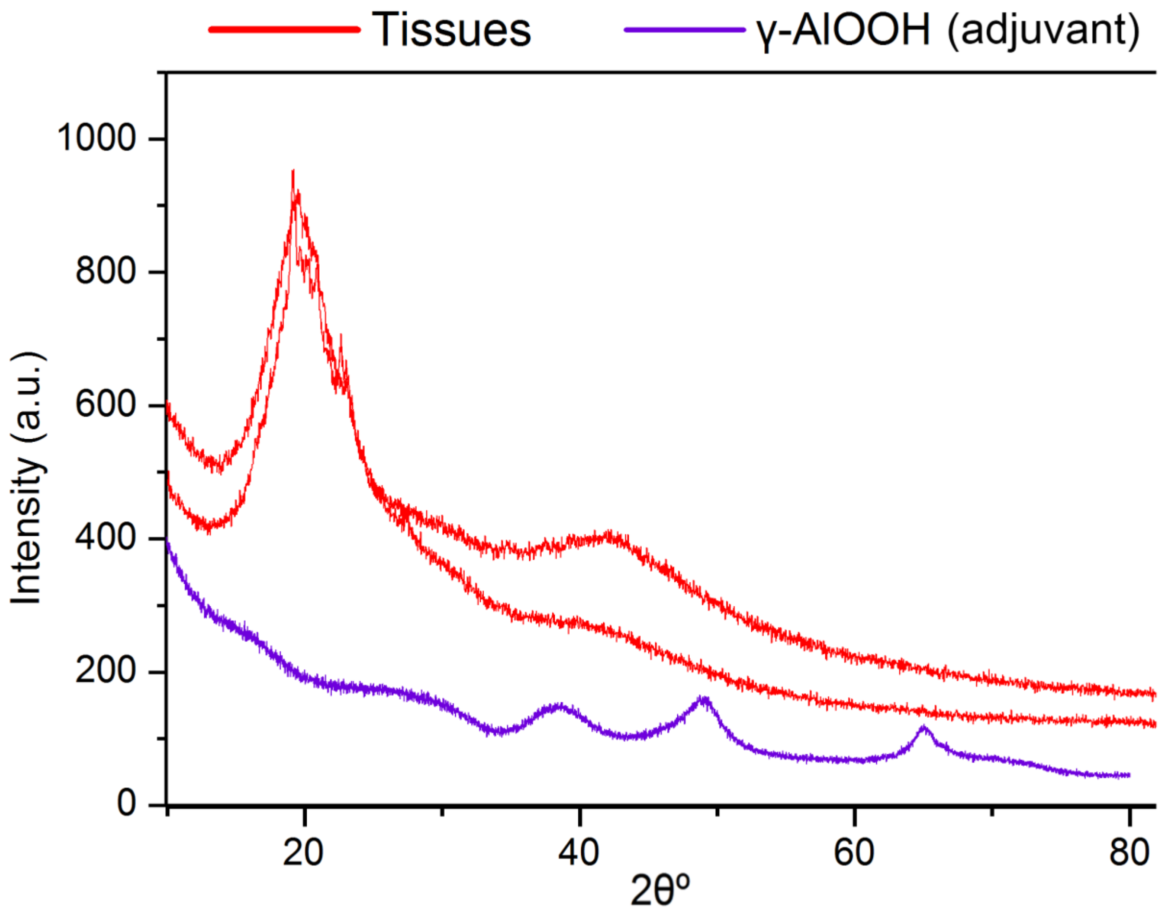

Figure S7. PXRD diffractograms of surface sections from two formalin-fixed granulomas in sheep showed higher overall intensity compared to the reference diffractogram of the γ-AlOOH adjuvant.

3. Supporting Tables

Table S1. Laser diffraction analysis of aluminum oxyhydroxide (AlOOH) adjuvant nanoparticles alone and in the vaccine formulation. Results are expressed as mean ± standard error of the mean.

Sample	Dv10 µm	Dv90 µm	Span
AlOOH M1	*M1*: 4.7 ± 0.1	*M1:* 16.7 ± 1.0	*M1:* 1.4 ± 0.1
AlOOH M2	*M2*: 22.4 ± 1.6	*M2:* 58.4 ± 11.5	*M2:* 1.0 ± 0.2
AlOOH M3	*M3*: 129.6 ± 26.8	*M3:* 296.7 ± 38.0	*M3*: 0.9 ± 0.1
Vaccine	15.0 ± 1.5	90.6 ± 7.6	2.1 ± 0.3

Dv10 and Dv90= 10 % and 90 % of the cumulative volume distribution; *M1, M2, M3*= mode 1, 2 and 3.

Table S2. SEM-EDS analysis. Complete unnormalized elemental composition of crystalloid bodies (CBs), aluminum aggregates (Al Aggr.), and background tissue. Data presented as mean ± standard deviation.

	**CBs (n=19)**		**Al Aggr. (n=7)**		**Background tissue (n=8)**	
	At.%	***Wt.%***	***At.%***	***Wt.%***	***At.%***	***Wt.%***
**Al**	1.8 ± 1.1 (19/19; 100 %)	3.3 ± 2.1 (19/19; 100 %)	0.8 ± 0.3 (7/7; 100 %)	1.6 ± 0.6 (7/7; 100 %)	0 (0 %)	0 (0 %)
**O**	26.3 ± 6.0 (19/19; 100 %)	29.6 ± 6.0 (19/19; 100 %)	17.5 ± 4.4 (7/7; 100 %)	21.2 ± 4.6 (7/7; 100 %)	15.6 ± 9.0 (8/8; 100 %)	18.8 ± 9.0 (8/8; 100 %)
**C**	69.9 ± 7.2 (19/19; 100 %)	59.8 ± 8.4 (19/19; 100 %)	79.7 ± 5.8 (7/7; 100 %)	72.1 ± 6.8 (7/7; 100 %)	83.6 ±8.4 (8/8; 100 %)	77.6 ± 11.5 (8/8; 100 %)
**P**	1.1 ± 8.4 (19/19; 100 %)	2.3 ± 1.4 (19/19; 100 %)	0.5 ± 0.4 (7/7; 100 %)	1.1 ± 0.4 (7/7; 100 %)	0 (0 %)	0 (0 %)
**Ca**	0.3 ± 0.1 (4/19; 21.05 %)	0.7 ± 0.2 (4/19; 21.1 %)	0 (0 %)	0 (0 %)	0 (0 %)	0 (0 %)
**Cl**	0.7 ± 0.5 (19/19; 100 %)	1.9 ± 1.2 (19/19; 100 %)	0.9 ± 0.4 (7/7; 100 %)	2.1 ± 0.5 (7/7; 100 %)	0 (0 %)	0 (0 %)
**U**	0.6 ± 1.8 (17/19; 84.47 %)	3.3 ± 1.7 (17/19; 89.5 %)	2.6 (1/7; 14.3 %)	3.2 (1/7; 14.29 %)	0.3 ± 0.0 (3/8; 37.5 %)	4.3 ± 0.6 (3/8; 37.5 %)
**Os**	0.1 (1/19; 5.3 %)	1.3 (1/19; 5.3 %)	0 (0 %)	0 (0 %)	0 (0 %)	0 (0 %)

CBs= crystalloid bodies; *Al Aggr.*= aluminum aggregates; *At.%*= atomic percentage; *Wt.%*= weight percentage; *Al*= aluminum; *O*= oxygen; *C*= carbon; *P*= phosphorus; *Ca*= calcium; *Cl*= chlorine; *U*= uranium; *Os*= osmium.

Table S3. Statistical comparison of atomic percentage (At.%) ratios from SEM-EDS analyses of crystalloid bodies (CBs), intracytoplasmic aluminum aggregates (Al aggr.), and background tissue (excluding cell nuclei).

Ratio (At.%)	Phase	Mn ± SD	Md ± IQR	CBs (p_{MW})	Al Aggr. (p_{MW})	Tissue (p_{MW})	p_{KW}
**Al:C**	CBs (n=19)	0.03 ± 0.02	0.02 ± 0.03	-	**0.04***	**<0.001*****	**<0.001*****
	Al Aggr (n=7)	0.01 ± 0.01	0.01 ± 0.01	**0.04***	-	**<0.001*****	
	Tissue (n=8)	0	0	**<0.001*****	**<0.001*****	-	
**Al:P**	CBs (n=19)	0.45 ± 0.78	1.56 ± 0.40	-	0.364	**<0.001*****	**<0.001*****
	Al Aggr (n=7)	1.56 ± 0.38	1.86 ± 0.48	0.364	-	**<0.001*****	
	Tissue (n=8)	0	0	**<0.001*****	**<0.001*****	-	
**P:C**	CBs (n=19)	0.02 ± 0.02	0.01 ± 0.02	-	**0.035***	**<0.001*****	**<0.001*****
	Al Aggr (n=7)	0.01 ± 0.01	0.004 ± 0.002	**0.035***	-	**<0.001*****	
	Tissue (n=8)	0	0	**<0.001*****	**<0.001*****	-	
**Al:O**†	CBs (n=19)	0.06 ± 0.03	0.06 ± 0.06	-	n.d.	n/d	0.193
	Al Aggr (n=7)	0.05 ± 0.01	0.05 ± 0.01	n.d.	-	n/d	
	Tissue (n=8)	0	0	n.d.	n.d.	-	n.d.

Ratio (At.%)= ratio calculated from atomic percentages; *Mn*= mean; *SD*= standard deviation, *Md*= median; *IQR*= interquartile range; *CBs*= crystalloid bodies; *Al Aggr.*= aluminum aggregates; p_{MW}= *p*-value of Mann-Whitney *U* test; p_{KW}= *p*-value of Kruskal-Wallis test; *Al:C*= aluminum-to-carbon ratio; *Al:P*= aluminum-to-phosphorus ratio; *P:C*= phosphorus-to-carbon ratio; n.d. = not determined; **p* < 0.05, ***p* < 0.01, ****p* < 0.001.

†Overall Mann-Whitney test applied to CBs and Al Aggr only; no post hoc pairwise rank analyses were performed.

Table S4. Correlation analysis of atomic percentage (At.%) ratios from SEM-EDS analyses of crystalloid bodies (CBs) and intracytoplasmic aluminum aggregates (Al aggr.).

**Ratio** (At.%)	**Mn ± SD**	**Md ± IQR**	**Al:C (CC/$p_{Rho\text{-}S}$)**	**Al:P (CC/$p_{Rho\text{-}S}$)**	**P:C (CC/$p_{Rho\text{-}S}$)**
**CBs (n=19)**					
**Al:C**	0.03 ± 0.02	0.02 ± 0.03	1.000/-	0.50/**0.029***	0.919/**<0.001*****
**Al:P**	0.45 ± 0.78	1.56 ± 0.40	0.50/**0.029***	1.000/-	0.458/**0.049***
**P:C**	0.02 ± 0.02	0.01 ± 0.02	0.919/**<0.001*****	0.458/**0.049***	1.000/-
**Al Aggr. (n=7)**					
**Al:C**	0.01 ± 0.005	0.01 ± 0.005	1.000/-	1.000/-	0.821/**0.023***
**Al:P**	1.56 ± 0.38	1.86 ± 0.48	0.000/1.000	1.000/-	-0.321/0.482
**P:C**	0.01 ± 0.005	0.004 ± 0.002	0.821/**0.023***	-0.321/0.482	1.000/-

At.%= atomic percentage; *CBs* =crystalloid bodies; *Al Aggr* = aluminum aggregates; *Al:C*= aluminum-to-carbon ratio; *Al:P*= aluminum-to-phosphorus ratio; *P:C*= phosphorus-to-carbon ratio; *Mn* = mean; *SD*= standard deviation; *Md*= median; *IQR*= interquartile range; *CC* = correlation coefficient; $p_{Rho\text{-}S}$= *p*-value from Spearman's rho correlation test; $*p < 0.05$, $**p < 0.01$, $***p < 0.001$.

Table S5. Statistical comparison of atomic (At.%) ratios between crystalloid bodies (CBs) with high electron signal intensity and detectable calcium (Ca), and CBs with low electron signal intensity and without detectable Ca.

Ratio (At.%)	Electron signal	Mean ± SD	Min	Q1	Q2	Q3	Max	p_{MW}
**Al:C**	High with Ca	0.45 ± 0.78	0.04	0.05	0.06	1.58	1.62	**0.001** **
	Low with no Ca	0.02 ± 0.01	0.00	0.01	0.02	0.04	0.04	
**Al:P**	High with Ca	1.52 ± 0.09	1.40	1.47	1.53	1.58	1.62	0.596
	Low with no Ca	1.29 ± 0.69	0.17	0.93	1.35	1.73	2.48	
**P:C**	High with no Ca	0.03 ± 0.01	0.02	0.03	0.03	0.03	0.04	**0.006** **
	Low with no Ca	0.01 ± 0.01	0.00	0.01	0.01	0.01	0.04	

At.%= atomic percentage; *Al:C*= aluminum-to-carbon ratio; *Al:P*= aluminum-to-phosphorus ratio; *P:C*= phosphorus-to-carbon ratio; *SD*= standard deviation; *Min*= minimum; *Q1*= first quartile; *Q2*= second quartile (median); *Q3*= third quartile; *Max*= maximum; p_{MW}= p-value of Mann-Whitney *U* test; *$p < 0.05$, **$p < 0.01$, ***$p < 0.001$.

Table S6. Correlation analysis of atomic (At.%) ratios between crystalloid bodies (CBs) with high electron signal intensity and detectable calcium (Ca), and CBs with low electron signal intensity and without detectable Ca.

Electron signal	Ratio (At.%)	Mean ± SD	Md ± IQR	Al:C (CC/p_{Rho-S})	Al:P (CC/p_{Rho-S})	P:C (CC/p_{Rho-S})
High with Ca (n=4)	**Al:C**	0.45 ± 0.78	0.06 ± 1.53	1.00/-	-0.80/0.20	1.00/-
	**Al:P**	1.52 ± 0.09	1.53 ± 0.11	-0.80/0.20	1.00/-	-0.80/0.20
	**P:C**	0.03 ± 0.01	0.03 ± 0.00	1.00/-	-0.80/0.20	1.00/-
Low with Ca (n=15)	**Al:C**	0.02 ± 0.01	0.02 ± 0.03	1.00/-	1.00/0.20	0.99/**<0.001*****
	**Al:P**	1.29 ± 0.69	1.35 ± 0.80	-0.36/0.20	1.00/-	-0.40/0.14
	**P:C**	0.01 ± 0.01	0.01 ± 0.00	0.99/**<0.001*****	-0.40/0.140	1.00/-

At.%= atomic percentage; *Al:C*= aluminum-to-carbon ratio; *Al:P*= aluminum-to-phosphorus ratio; *P:C*= phosphorus-to-carbon ratio; *SD*= standard deviation; *Md*= median; *IQR*= interquartile range; *CC* = correlation coefficient; p_{Rho-S}= *p*-value from Spearman's rho correlation test; *$p < 0.05$, **$p < 0.01$, ***$p < 0.001$.

Table S7. TEM-EDS analysis of crystalloid bodies (CBs), aluminum aggregates (Al Aggr.), and background tissue. Complete unnormalized elemental composition. Data are presented as mean ± standard deviation.

	**CBs (n=7)**		**Al Aggr. (n=6)**		**Background tissue (n=6)**	
	At.%	*Wt.%*	*At.%*	*Wt.%*	*At.%*	*Wt.%*
**Al**	4.2 ± 5.6 (100 %)	5.9 ± 7.0 (100 %)	1.9 ± 0.9 (100 %)	3.8 ± 1.7 (100 %)	0.0 ± 0.0 (100 %)	0.0 ± 0.0 (100 %)
**O**	26.2 ± 15.2 (100 %)	22.7 ± 10.5 (100 %)	12.3 ± 3.0 (100 %)	14.5 ± 3.2 (100 %)	3.0 ± 2.4 (100 %)	3.8 ± 3.0 (100 %)
**C**	52.5 ± 30.9 (100 %)	40.2 ± 0.3 (100 %)	83.9 ± 3.7 (100 %)	73.7 ± 5.4 (100 %)	94.5 ± 2.7 (100 %)	88.9 ± 2.2 (100 %)
**P**	1.1 ± 2.1 (71%)	2.0 ± 3.9 (71 %)	0.5 ± 0.3 (83 %)	1.1 ± 0.6 (83 %)	0.0 ± 0.0 (17 %)	0.1 ± 0.1 (17 %)
**Ca**	0.7 ± 0.3 (43 %)	1.7 ± 4.0 (43 %)	0.0 ± 0.0 (33 %)	0.0 ± 0.1 (33 %)	0.0 ± 0.0 (17 %)	0.1 ± 0.1 (100 %)
**N**	1.0 ± 1.1 (57 %)	0.8 ± 0.9 (57 %)	1.2 ± 1.7 (67 %)	1.3 ± 1.8 (67 %)	1.6 ± 1.1 (100 %)	0.9 ± 1.2 (100 %)
**Cl**	0.0 ± 0.0 (14 %)	0.0 ± 0.0 (14 %)	0.0 ± 0.0 (17 %)	0.0 ± 0.1 (17 %)	15.6 ± 9.0 (66.7 %)	15.6 ± 9.0 (66.7 %)
**Na**	0.2 ± 0.4 (100 %)	0.3 ± 0.5 (100 %)	15.6 ± 9.0 (100 %)	15.6 ± 9.0 (100 %)	15.6 ± 9.0 (100 %)	15.6 ± 9.0 (100 %)
**Si**	9.7 ± 14.1 (100 %)	13.5 ± 19.0 (100 %)	15.6 ± 9.0 (100 %)	15.6 ± 9.0 (100 %)	15.6 ± 9.0 (100 %)	15.6 ± 9.0 (100 %)
**S**	0.1 ± 0.2 (57 %)	0.2 ± 0.3 (57 %)	0.0 ± 0.1 (33 %)	0.0 ± 0.1 (33 %)	0.0 ± 0.0 (100 %)	0.1 ± 0.0 (100 %)
**K**	1.0 ± 2.1 (57.1 %)	1.9 ± 3.8 (57 %)	0.2 ± 0.2 (50 %)	0.5 ± 0.7 (50 %)	0.0 ± 0.0 (17 %)	0.0 ± 0.1 (17 %)
**Mg**	0.3 ± 0.4 (57 %)	0.4 ± 0.5 (57 %)	0.0 ± 0.0 (100 %)	0.1 ± 0.2 (100 %)	0.1 ± 0.1 (17 %)	0.1 ± 0.2 (17 %)
**Fe**	0.2 ± 0.3 (86 %)	0.3 ± 0.4 (100 %)	0.0 ± 0.0 (33 %)	0.0 ± 0.1 (33 %)	0.0 ± 0.0 (100 %)	0.0 ± 0.0 (100 %)
**Cu**	2.7 ± 2.7 (100 %)	9.3 ± 10.0 (100 %)	1.0 ± 0.7 (83 %)	5.3 ± 1.7 (83 %)	1.1 ± 0.3 (100 %)	5.3 ± 1.7 (100 %)
**V**	0.0 ± 0.0 (14%)	0.0 ± 0.0 (14.3 %)	0.0 ± 0.0 (100 %)	0.0 ± 0.0 (100 %)	0.0 ± 0.0 (100 %)	0.0 ± 0.0 (100 %)

CBs= crystalloid bodies; *Al Aggr.*= aluminum aggregates; *At.%*= atomic percentage; *Wt.%*= weight percentage; *Al*= aluminum; *O*= oxygen; *C*= carbon; *P*= phosphorus; *Ca*= calcium; *N* = nitrogen; *Cl*= chlorine; *Na* = sodium; *Si* = silica; *S* = sulfur; *K* = potassium; *Mg* = magnesium; *Fe* = iron; *Cu* = copper; *V* = vanadium.

Table S8. Detailed breakdown of all elements detected in background tissue (phase 1), crystalloid bodies (CBs, phase 2), and aluminum nanoparticles (phase 3), as identified by SEM-ChemiPhase analysis using the Thermo Scientific™ Apreo™ ChemiSEM system.

Elem.	**P1-*Background***		**P2-*CBs***		**P3-*Al nanoparticles***	
	At.%	*Wt.%*	*At.%*	*Wt.%*	*At.%*	*Wt.%*
**C**	70.1	61.5	61.2	50.0	66.9	56.9
**P**	0.1	0.2	1.1	2.2	25.2	28.6
**N**	4.1	4.2	5.0	4.8	6.2	6.2
**O**	24.6	28.8	29.7	32.3	25.2	28.6
**Al**	0.2	0.4	2.0	3.7	0.4	0.7
**Ca**	-	-	0.1	0.3	-	0.1
**Si**	0.0	0.1	0.0	0.1	0.0	0.1
**S**	0.0	0.1	0.0	0.1	0.0	0.1
**Cl**	0.7	1.9	0.7	1.7	0.7	1.9
**U**	0.1	1.7	0.2	3.1	0.1	3.1
**Os**	0.1	1.1	0.2	2.1	0.2	2.1

Elem= element; *Background*= rest of tissue areas lacking CBs and Al nanoparticles; *CBs*= crystalloid bodies; *At.%*= atomic percentage; *Wt.%*= weight percentage.
